# A branching stochastic evolutionary model of the B-cell repertoire

**DOI:** 10.1007/s00285-024-02102-y

**Published:** 2024-06-07

**Authors:** Ollivier Hyrien, Nikolay M. Yanev

**Affiliations:** 1 Biostatistics, Bioinformatics, Epidemiology Program, Vaccine and Infectious Disease Division, Fred Hutchinson Cancer Center, Seattle, WA USA; 2 Department of Operations Research, Probability and Statistics, Institute of Mathematics and Informatics, Bulgarian Academy of Sciences, Sofia, Bulgaria

**Keywords:** Multitype branching process, Temporal alpha and beta diversity, Convergent evolution, Germinal center, Sequential immunization, 60J80, 60J85

## Abstract

We propose a stochastic framework to describe the evolution of the B-cell repertoire during germinal center (GC) reactions. Our model is formulated as a multitype age-dependent branching process with time-varying immigration. The immigration process captures the mechanism by which founder B cells initiate clones by gradually seeding GC over time, while the branching process describes the temporal evolution of the composition of these clones. The model assigns a type to each cell to represent attributes of interest. Examples of attributes include the binding affinity class of the B cells, their clonal family, or the nucleotide sequence of the heavy and light chains of their receptors. The process is generally non-Markovian. We present its properties, including as $t\rightarrow \infty$ when the process is supercritical, the most relevant case to study expansion of GC B cells. We introduce temporal alpha and beta diversity indices for multitype branching processes. We focus on the dynamics of clonal dominance, highlighting its non-stationarity, and the accumulation of somatic hypermutations in the context of sequential immunization. We evaluate the impact of the ongoing seeding of GC by founder B cells on the dynamics of the B-cell repertoire, and quantify the effect of precursor frequency and antigen availability on the timing of GC entry. An application of the model illustrates how it may help with interpretation of BCR sequencing data.

## Introduction

During an infection, the immune system initiates a response to protect the body from the invading pathogen and establish immunity against future reinfections. This response often relies on B cells, a class of lymphocytes specialized in the production of antibodies that neutralize invaders by binding to foreign target molecules on the pathogen called *antigens*. Since each antibody binds to specific molecules, a highly diverse B-cell repertoire is essential for achieving robust immune protection. While naive B cells express antibodies with considerable molecular diversity due to somatic recombination (Dreyer and Bennett 1965; Tonegawa 1983), the B-cell repertoire must undergo additional diversification to effectively respond to a vast array of evolving pathogens (Janeway et al. 2005).

The B-cell repertoire further diversifies during affinity maturation, the Darwinian evolutionary process that occurs in germinal centers (GC) (Janeway et al. 2005; MacLennan 1994; De Silva and Klein 2015). GC are temporary structures that develop in secondary lymphoid tissues such as lymph nodes, spleen, tonsils, and Peyer’s patches in the gut. They provide the microenvironment that supports and regulates adaptation of the B-cell repertoire to the invading pathogen. They are continually seeded by B cells selected for their ability to bind to the antigen (Schwickert et al. 2007). They include two distinct anatomical compartments, the dark and light zones, between which B cells traffic back and forth to undergo successive rounds of proliferation and somatic hypermutation followed by antigen-mediated selection (Eisen and Siskind 1964; Weigert et al. 1970; Jacob et al. 1991; Muramatsu et al. 2000). Somatic hypermutation occurs at an extraordinarily high rate estimated at $\sim 2\times 10^{-4} - 10^{-3}$ per base pair per generation which is about a million times greater than the mutation rate observed in other parts of the genome (Berek and Milstein 1987; McKean et al. 1984).

Understanding the rules of affinity maturation has major clinical applications. For example, several clinical trials currently in progress are testing novel HIV vaccines that seek to harness this process to elicit B cells able to secrete broadly neutralizing antibodies (bnAbs) similar to those isolated in people living with HIV (Leggat et al. 2022). By deciphering the rules governing antibody maturation, immunologists could manipulate GC to devise effective *immunogens* (i.e., molecules capable of inducing an immune response) and vaccination strategies aimed at preventing HIV acquisition and infection against other pathogens.

Mathematical models have played a crucial role in advancing our understanding of the dynamics of GC B cells, shedding light on this complex evolutionary system. Notably, pioneering work in the field can be found in (Agur et al. 1991; Kepler and Perelson 1993a, b, 1995; Oprea and Perelson 1997; Kleinstein and Singh 2001; Meyer-Hermann et al. 2001; Iber and Maini 2002) while a recent review is provided in Buchauser and Wadermann (2019). Many of these models are formulated as agent-based models that evaluate properties of GC via simulations. While this approach considers the stochastic nature of GC reactions, it may be computer-intensive and only yield conclusions for chosen parameter values. In this paper, we propose a comprehensive stochastic framework that allows for a more systematic exploration of properties across a range of plausible parameter values. We demonstrate the utility of this framework by showing that it predicts established properties of GC, while offering explanations for these properties and enabling the identification of new ones.

Somatic hypermutation and the competition between GC B cells are two central factors that structure evolution and adaptation of the B-cell repertoire. We propose a model that captures the most salient features of the process by assuming that the dynamics of GC B cells is controlled by three mechanisms: (1) the continual recruitment of founder B cells; (2) clonal expansion; and (3) somatic hypermutation of the BCR-encoding immunoglobulin (Ig) gene loci.

The continual recruitment of founder B cells induces an influx of B cells into GC and initiates the formation of clones (Schwickert et al. 2007, 2009; Turner et al. 2017). We model this mechanism using a counting process, potentially time-inhomogeneous. Evolution of B-cell clones is driven by cellular division, death, and differentiation, which we describe using an age-dependent branching process. Finally, accumulation of somatic hypermutations, the mechanism which randomly alters the binding properties of BCR, is captured by assigning a *type* to each cell and defining a network specifying the set of admissible transitions between types as cells divide. It is important to note that types need not represent nucleotide or amino acid sequences of BCR, but could instead reflect associated properties such as binding affinity classes or clonal families. Differences in the probability of death between types capture differential fitness (competition) between different types of B cells. The resulting model is a multitype age-dependent branching process with immigration, potentially time-varying, extending previously considered models (e.g., Sevastyanov 1957; Jagers 1968; Hyrien et al. 2005, 2017, 2015; Pakes and Kaplan 1974; Mitov et al. 2018; Slavtchova-Bojkova et al. 2023a, b; Yakovlev and Yanev 2006). The model allows tailored network architectures to study evolution of a variety of features of the BCR repertoire. Numerical methods have been proposed to implement these models (e.g., saddlepoint approximations to moments Hyrien et al. 2010).

We demonstrate the flexibility of the framework by studying three model structures. The first one classifies B cells according to their binding affinity for a given *antigen* (see Sect. 3.5.1). We show that, as a class, lower binding affinity B cells have a competitive advantage over their higher-affinity counterparts because of their propensity to engage in GC reactions earlier, attributed to their higher abundance. We also find that clonal families should contain B cells with diverse binding affinity levels that grow in size at the same rate within their respective clones, a finding aligning with previous observations (Kuraoka et al. 2016; Bannard and Cyster 2017). No definitive mechanisms have been put forth to explain this phenomenon; our model shows that it could be, in part, attributed to the combined effects of clonal expansion and somatic hypermutation (Sect. 3.7). Mathematically, this property emerges from the communication between cell types (or, equivalently, the irreducibility of the mean offspring matrix). From a biological standpoint, it results from the fact that somatic hypermutation may alter affinity for the antigen, and that these alterations can be reversed.

Several studies have shown that early GC harbor B cells from multiple clonal families, potentially hundreds, and gradually loose this clonal diversity to become *oligoclonal* (i.e., dominated by a few clones) (Kroese et al. 1987; Janeway et al. 2005; Faro and Or-Guil 2013; Tas et al. 2016). To study evolution of clonal dominance, we consider a second model in which each type identifies a clonal family. Analysis of the model supports that oligoclonality arises from differential binding affinity between clones. It also suggests that clonal dominance may be a temporary feature repeatedly lost to newer clones until the GC reaction stops (see Sects. 3.5.2, 5).

**Fig. 1:**
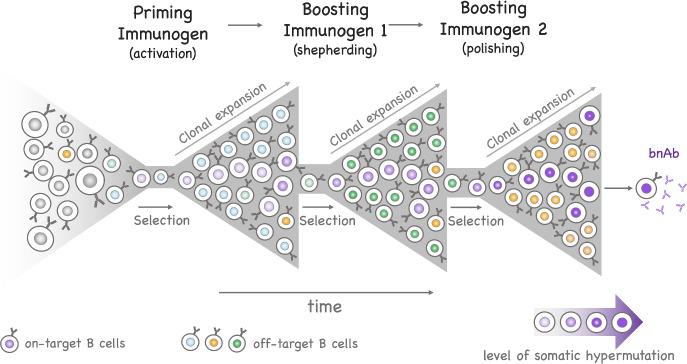
*Principle of a successful sequential immunization strategy.* The vaccine regimen begins with a priming immunogen seeking to induce B cells with genetic and molecular signatures expected to allow HIV bnAb production. B cells induced by the prime consist of both desired and non-desired (on- and off-target) B cells that express BCR with limited somatic hypermutations and lack the ability to neutralize the virus. To induce B cells with neutralizing activities, the immune system is boosted by a series of immunogens designed to support further maturation of B cells from clonal families induced by previous vaccine doses and select somatic hypermutations relevant for broad neutralization against HIV. After each vaccination, on-target B cells compete with off-target B cells for antigen-mediated selection

We introduce a third model designed to examine the divergence of BCR sequences from their unmutated (germline) ancestors, driven by the accumulation of somatic hypermutations. This model is relevant for characterizing the evolution of the B-cell repertoire in the context of immunization. Most vaccines achieve protection through induction of antibodies (Plotkin 2008). Protective vaccines against the human immunodeficiency virus (HIV) have remained elusive to date. However, about 20–30% of people with HIV infection develop bnAbs which neutralize many of the globally circulating HIV strains through binding conserved regions of the virus (Doria-Rose et al. 2009). VRC01 is one bnAb that specifically targets the CD4 binding site on the HIV virus. The efficacy of passive immunization with VRC01 against HIV acquisition was recently tested in two clinical trials which showed that protection against HIV infection by VRC01 was confined to viruses highly susceptible to neutralization (Corey et al. 2021). Novel vaccines that seek to induce HIV bnAbs via sequential immunization are currently being evaluated (Haynes and Mascola 2017; Leggat et al. 2022) (see Fig. 1 for a description of their principle). One challenging goal of this strategy is selection of somatic hypermutations similar to those of HIV bnAbs, including rare substitutions, capable of neutralizing the virus. One metric for assessing the vaccine-induced immune response is the distance between the sequences of vaccine-elicited BCR to that of a designated target bnAb. The third model describes the progression of these distances over time (Sect. 6).

**Fig. 2:**
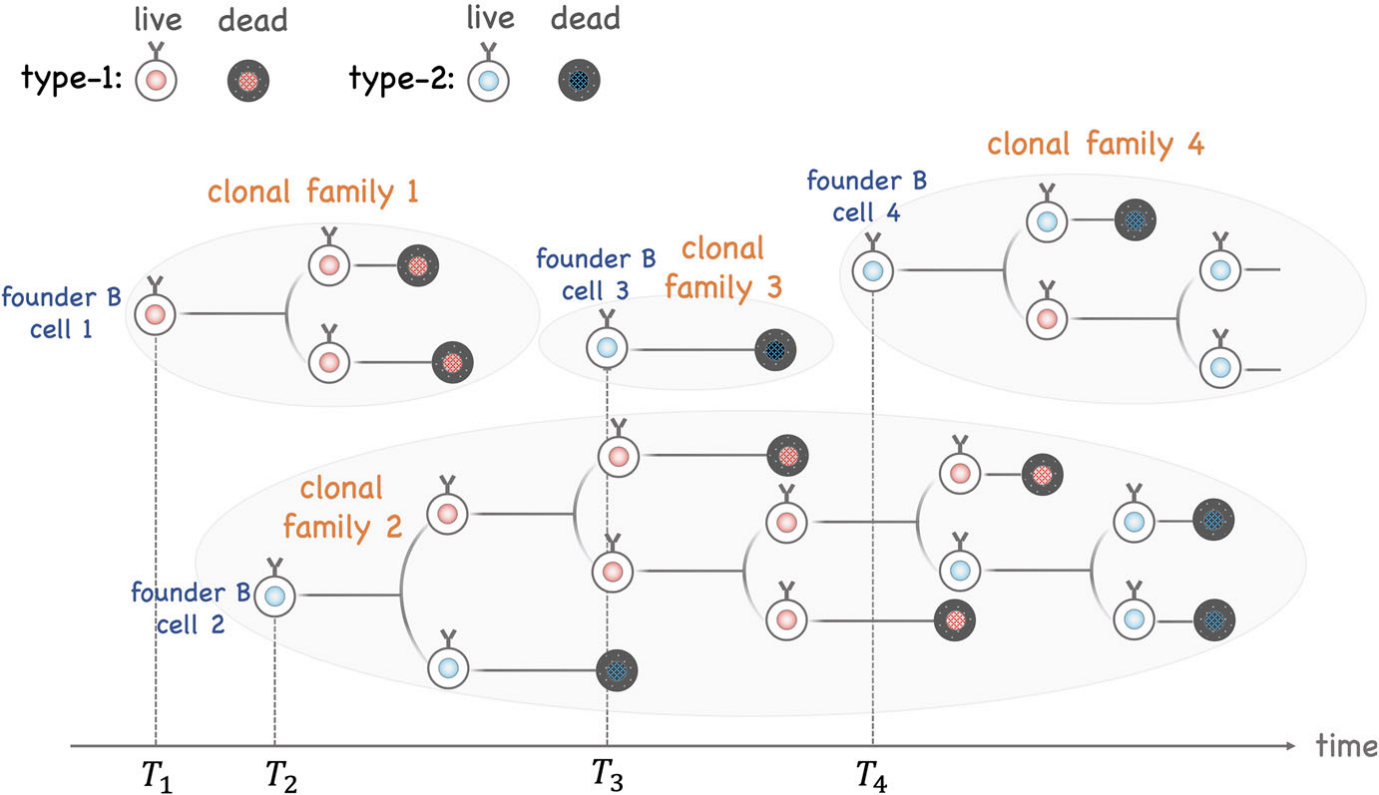
*Dynamics of germinal center B cells as captured by the proposed model.* Founder B cells join the GC at random time points ($T_1, T_2, T_3, T_4 \ldots$). These arrival times are described by a point process, potentially time-inhomogeneous. Each founder B cell initiates a clonal family in which B cells either divide or die. Each cell is assigned a type at birth; when it divides it may produce offspring of different types (e.g., reflecting the impact of somatic hypermutations). In the example depicted here, the population includes two types of B cells, but more complex models with arbitrary number of types may be constructed

The remainder of this paper is organized as follows. Section 2 provides a brief review of how antibodies are encoded and their structure. The general model is defined in Sect. 3; see Fig. 2 for a graphical overview. Section 3.2 discusses the model that describes the seeding of GC. Section 3.3 focuses on the particular case where this model is a time-inhomogeneous Poisson process. Section 3.4 presents the multitype branching process that describes clonal expansion and the impact of somatic hypermutation. The specific models introduced earlier are defined in Sect. 3.5. Model properties are presented in Sects. 3.6, 5 and 6 to explore patterns of the dynamics of GC reactions. Our study of clonal dominance relies on measuring the alpha and beta diversity of B-cell repertoires; these indices are presented in Sect. 4 in the general context of multitype branching processes. Throughout, results are discussed and illustrated in the context of sequential immunization, introduced in Sect. 6.1. Technical details (proofs) are provided in the Appendix.

## Encoding and structure of antibodies

**Fig. 3:**
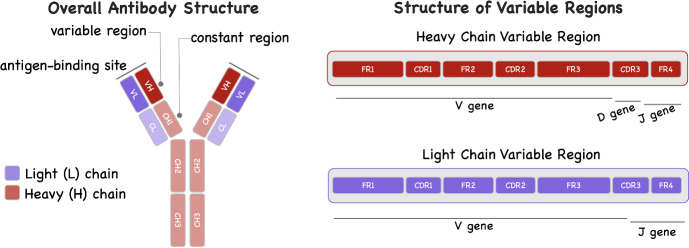
Structure of antibodies; see Sect. 2 for details

Antibodies consist of four polypeptide chains arranged in two identical pairs, with each pair consisting of a heavy and a light chain connected by disulfide bonds (Fig. 3). The stem of the (Y-shaped) antibody molecule, called the constant region, determines the class of the antibody (IgG, IgA, IgM, IgD, and IgE). The tips of the antibody molecule are known as the antigen-binding regions or variable regions. The variable regions of the antibody molecule contains regions called complementarity-determining regions (CDR). There are three CDRs in both the variable heavy and variable light chains, denoted as CDR1, CDR2, and CDR3. These loops are the most variable parts of the antibody, and their unique sequences are responsible for antigen recognition. The unique sequence of amino acids in the CDRs allows each antibody to bind to a specific target antigen or a closely related group of antigens. Between the CDRs, there are four regions known as framework regions (FR1-FR4) which provide the structural scaffold for the variable region. While they are less variable than the CDRs, variations in the nucleotide sequence of the FRs can still affect the antigen-binding properties of the antibodies. The combination of the CDRs and the adjacent FRs forms the antigen-binding site of the antibody.

The genetic information encoding the variable regions is divided into multiple variable (V), diversity (D, only for heavy chains), and joining (J) gene segments. During B cell development, the DNA of the B cell randomly rearranges one gene segment from each V(D)J category per chain. The selected gene segments are joined together, and any excess DNA between them is removed. This process which takes place in the bone marrow is called somatic recombination. It results in a functional gene encoding the variable region of the antibody. Variable regions are approximately 110–130 amino acid long in heavy chains, and slightly shorter in light chains. Most positions are encoded by the V gene.

After the B cell encounters an antigen, it may undergo somatic hypermutation in the variable regions of its Ig genes. This process introduces random mutations into these genes, primarily in the CDRs. B cells with mutated antibodies that bind more effectively to the antigen receive a survival advantage and are selected for further proliferation, eventually leading to the production of high-affinity antibodies. Somatic hypermutation further improves these antibodies for optimal antigen recognition. This process produces more effective antibodies over time during an immune response. See Janeway et al. (2005) for further details.

## Stochastic modeling of GC B-cell dynamics

### Classification of B cells into types

All BCR expressed on the surface of a B cell (around 120,000; Alt et al. 2015, p. 154) share the same nucleotide sequence that determines their antigen specificity (Janeway et al. 2005; Alt et al. 2015). During a GC reaction, proliferating B cells accumulate somatic hypermutations in their Ig gene loci, causing BCR sequences to drift away from the original (naive) sequence assembled during somatic recombination. This evolutionary process, which enables adaptation of the B-cell repertoire, may be studied from many angles. Aside from the sequence of the B-cell receptors, relevant properties (attributes) include their binding affinity class and clonal family. We propose a unified modeling framework in which a generic type is assigned to each B cell. These types are indexed by a set $\mathcal {K}$, and we let $K = \vert \mathcal {K} \vert$ denote the total number of types, possibly infinite, in the model. Types represent arbitrary attributes, but it may be convenient to identify them by the first *K* integers and set $\mathcal {K}=\{1,\ldots ,K\}$ when useful. Every B cell is assumed to belong to one of these *K* types which induce a partition of the B-cell repertoire.

### Seeding of germinal centers as a counting process

Schwickert et al. (2007) experimentally showed that GC are open structures that are continually visited and seeded by naive follicular B cells (Schwickert et al. 2007). We describe the ongoing seeding of GC using *K* type-specific immigration processes, one per cell type. These processes are formulated by defining *K* sequences of positive and increasing random variables (r.v.) $\{T_{k\ell },\ell =1,2\ldots \}$ where $0\le T_{k1} \le T_{k2} \cdots$ a.s., $k\in \mathcal {K}$. Each of these sequences represents the successive time points at which type-*k* founder B cells join the GC reaction. The first type-*k* founder B cell enters the GC at time $T_{k1}$, the second one at time $T_{k2}$, and so on. The sequence $\{T_{k\ell },\ell =1,2\ldots \}$ generates a counting process $\Pi _k(t) = \sum _{\ell =1}^\infty \textbf{1}_{\{T_{k\ell } \le t \}}$, $t \ge 0$, which represents the number of type-*k* founder B cells that have entered the GC by time *t*. Let $\{T_\ell ,\ell = 1,2\ldots \} = \bigcup _{k \in \mathcal {K}} \{T_{k\ell },\ell = 1,2\ldots \}$ denote the collection of all time points at which founder B cells enter the GC, and define the overall immigration process

1 $$\begin{aligned} \Pi (t) = \sum _{\ell =1}^\infty \textbf{1}_{\{T_{\ell } \le t \}} = \sum _{k \in \mathcal {K}} \Pi _k(t) \quad (t\ge 0), \end{aligned}$$

which represents the total number of founder B cells that have joined the GC by time *t*, regardless of type. The specification of the counting processes $\{\Pi _k(\cdot )\}_{k \in \mathcal {K}}$ is context-dependent. Given its importance in applications, the Poisson process is treated in detail in the next section. An example where they are not defined as Poisson processes is given in Sects. 3.5.2 and 5.

### A time-inhomogeneous Poisson race

Throughout this section, we assume that:

1. $\{\Pi _k(\cdot )\}_{k\in \mathcal {K}}$ are mutually independent Poisson processes with local intensities $r_k(t)>0$ and mean measures $R_k(t)=\int _{0}^{t}r_k(x)dx <\infty$, $t\ge 0$, $k \in \mathcal {K}$.

The rate at which founder B cells enter GC has not been fully elucidated, and each $\Pi _k(\cdot )$ could be formulated as a time-homogeneous Poisson process for simplicity. The ability of naive antigen-specific B cells to enter pre-existing GC is impacted by multiple factors, including antigen availability. Turner et al. (2017) showed that B cells that acquire a small amount of antigen can enter GC at any stage of the reaction; however, naive antigen-specific B cells may be recruited during a more restricted time window (within 6–10 days) (Turner et al. 2017). Taken together, these findings support specifying $\Pi _k(\cdot )$ as a time-inhomogeneous Poisson process with rate $r(\cdot )$ functionally related to antigen availability.

We refer to Resnick (1992) and Durrett (2004) for discussions on Poisson processes, and recall the following useful five facts about them:

- *Fact 1:* $$\textbf{P}\{\Pi _k(t) = n\} = {R_k(t)^n}e^{-R_k(t)}/{ n!}$$, $$n=0,1\ldots $$, $$k \in \mathcal {K}$$, $$t\ge 0$$.
- *Fact 2:* $$\textbf{E}(\Pi _k(t) ) = \text{ Var }(\Pi _k(t) ) = R_k(t)$$, $$k \in \mathcal {K}$$, $$t\ge 0$$.
- *Fact 3:* If $\Pi _1(\cdot )$ and $\Pi _2(\cdot )$ are independent, possibly time-inhomogeneous, Poisson processes with local intensities $r_1(\cdot )$ and $r_2(\cdot )$, then $\Pi _1(\cdot )+\Pi _2(\cdot )$ is a Poisson process with local intensity $r_1(\cdot )+r_2(\cdot )$.
- *Fact 4:* If $\Pi _1(\cdot )+\Pi _2(\cdot )$ jumps by one unit at time *t*, then the probability that the jump is due to $\Pi _1(\cdot )$ is $r_1(t)/(r_1(t)+r_2(t))$.
- *Fact 5:* The time of arrival of the first type-*k* founder in the GC, $T_{k1}$, is a continuous r.v. with probability density function (p.d.f.) $f_{k1}(t) = r_k(t) e^{-R_k(t)}$, $t\ge 0$, and expectation $\mu _{k1}= \int _0^\infty e^{- R_k(v) } dv$.

When *K* is finite, we deduce from Fact 3 that $\Pi (\cdot )$ is a Poisson process with local intensity $r(\cdot ) = \sum _{k \in \mathcal {K}} r_k(\cdot )$. When $K=\infty$, this property still holds if $R(t) = \sum _{k \in \mathcal {K}} R_k(t)<\infty$, $t\ge 0$.

To identify the type of the $\ell$-th founder, define a r.v. $\textbf{I}_\ell = (I_{\ell k},k \in \mathcal {K})$ where $I_{\ell k}$ denotes the number of type-*k* founder B cells that entered the GC at time $T_\ell$, $\ell =1,2\ldots$. We assume that a single B cell immigrates at each time $T_\ell$; hence, $I_{\ell k}\in \{0,1\}$ and $\sum _{k \in \mathcal {K}}I_{\ell k}=1$ with probability one. We also assume that $\{\textbf{I}_\ell \}_{\ell =1}^\infty$ are independent and identically distributed (i.i.d.) r.v. with p.g.f. $g(\textbf{s}) = \mathbb {E} \left( \textbf{s}^{\textbf{I}_\ell } \right) = \sum _{k\in \mathcal {K}}g_{k}s_{k}$ where $\textbf{s}^{\mathbf \alpha } = \prod _{k \in \mathcal {K}} {s}_k^{\alpha _k}$.

On occasion, we will make the following assumption:

(A2) There exists positive constants $\{g_k\}_{k\in \mathcal {K}}$ such that $\sum _{k\in \mathcal {K}} g_k=1$ and $r_k(t) = g_k r(t)$ for every $t\ge 0$ and $k\in \mathcal {K}$.

Under Assumption (A2), temporal fluctuations in the influx of founder B cells remain identical across types, up to the multiplicative constants $$g_k$$. Together with Fact 4, this implies that $$g_k = r_k(t)/\sum _{i \in \mathcal {K}}r_i(t)$$ is the probability that a founder B cell that joins the GC at time *t* is of type *k*. Assumption (A2) implies that these probabilities remain unchanged throughout time, even when $$r(\cdot )$$ is time-dependent. When each process $$\Pi _k(\cdot )$$, $$k\in \mathcal {K}$$, is a time-homogeneous Poisson process with rate $$ g_k r_0$$ for some constant $$r_0\in (0,\infty )$$, then $$\Pi (\cdot )$$ is a time-homogeneous Poisson process with rate $$r(\cdot ) \equiv r_0$$.

For every $k \in \mathcal {K}$, the r.v. $T_{k1}$ denotes the waiting time until the first type-*k* founder enters the GC. According to Fact 5, $T_{k1}$ has a distribution with hazard rate $r_k(\cdot )$. If immigration is time-homogeneous, with $r_k(t) = g_k r_0$, $t\ge 0$, then $T_{k1}\sim Exp( g_k r_0)$. If $r_k(t) = g_k r_0 t^{\gamma }$ for some constants $r_0>0$ and $\gamma >-1$, then $T_{k1}$ has a Weibull distribution with shape and scale parameters $1+\gamma$ and $\left( \frac{1+\gamma }{ g_k r_0 }\right) ^{\frac{1}{1+\gamma }}$, heavy tailed when the immigration rate decreases over time at a polynomial rate $\gamma \in (-1,0)$. Its median and mean are $\left( \frac{1+\gamma }{ g_k r_0 }\right) ^{\frac{1}{1+\gamma }} \ln (2)^{\frac{1}{1+\gamma }}$ and $\left( \frac{1+\gamma }{ g_k r_0 }\right) ^{\frac{1}{1+\gamma }} \Gamma \left( 1 + \frac{1}{1+\gamma } \right)$ where $\Gamma (z) = \int _0^\infty u^{z-1}e^{-u}du$, and the ratio of the medians or means of the waiting time until first type-*k* and first type-$k'$ founders join the GC are both given by $\left( {g_{k'}}/{g_{k}}\right) ^{\frac{1}{1+\gamma }}$. We note that the assumption of a decreasing immigration rate may partly reflect current vaccination strategies where doses are typically administered as a bolus.

**Fig. 4:**
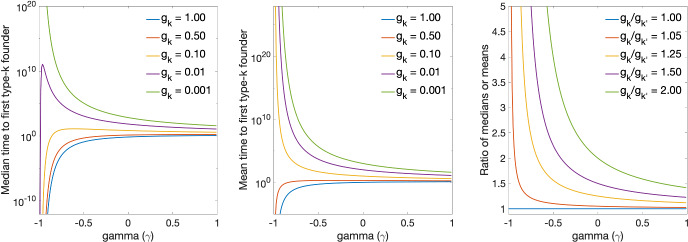
From left to right: median, mean, and ratio of medians (or means) of the time to first type-*k* founder joining the GC when the time-inhomogeneous Poisson process has local intensity $r_k(t)=g_k r_0 t^\gamma$ ($r_0=1$). These parameters are plotted as a function of $\gamma \in (-1,1)$

Figure 4 shows the median, mean, and ratio of medians or means of $T_{k1}$ as a function of $\gamma$, for various values of the precursor frequency $g_k$ and of $g_k/g_{k'}$. As $g_k$ decreases, both the median and mean increase. The median is neither a decreasing nor monotone function of $\gamma \in (0,\infty )$: it increases for $\gamma \in (-1,({g_k r_0e^{1}}-\ln 2)/{\ln 2})$ and decreases for $\gamma \in ({g_k r_0e^{1}}-\ln 2)/{\ln 2}, \infty )$. The ratio comparing the medians for two cell types (less versus more abundant) decreases for $\gamma \in (-1,\infty )$. When the influx of B cells recruited by the GC slows down ($\gamma \in (-1,0)$), the median waiting time is disproportionately longer for rarer cell types, and shorter when the influx accelerates. For instance, with an immigration rate decreasing at a rate of $\gamma =-1/2$ (i.e., $r_k(t)=g_k r_0 t^{-1/2}$), every halving of the frequency of the founder B cells (i.e., $g_k/g_{k'}=2$) causes the median or mean waiting time to increase by a factor of 4 ($=2^{\frac{1}{1-1/2}}$), compared to 2 ($=2^{\frac{1}{1-0}}$) when immigration is stationary ($r_k(t)=g_k r_0$, $\gamma =0$).

#### Remark 1

(*Biological relevance*) During an immune response, the recruitment of new B cells by GC may slow down when the amount of antigen that fuels the reaction decreases (Turner et al. 2017). The time until rare B cells (which tend to join the reaction later than more common ones, as discussed below and in Remark 2) join the GC may be longer than the waiting time for more common B cells. The fueling of GC by a continual supply of antigen, perhaps characteristic of chronic infections or GC in the gut, could increase the likelihood of rare B cells entering GC. This observation has clinical applications, particularly for the development of vaccines that seek to elicit responses from B cells with rare genetic and molecular signatures which could benefit from sustained immunogen delivery. A related phenomenon has been recently reported (Lee et al. 2022).

The recruitment of founder B cells into GC can be cast as a competition between classes of B cells that race against each other to place some of their members into the GC reaction. Let

$$\begin{aligned} N(\mathcal {K}_1,\mathcal {K}_2):= \sum _{k_1 \in \mathcal {K}_1}\Pi _{k_1} \big ( \min _{k_2 \in \mathcal {K}_2} T_{k_21} \big ) \end{aligned}$$

denote the number of type-$k_1$ founder B cells, $k_1 \in \mathcal {K}_1$, that have joined a given GC by the time the first type-$k_2$ founder B cell, $k_2 \in \mathcal {K}_2$, joins the same GC; this time is $\min _{k_2 \in \mathcal {K}_2} T_{k_21}$.

Suppose that $\mathcal {K}_1$ and $\mathcal {K}_2$ are two non-overlapping subsets of $\mathcal {K}$, that Assumption (A2) holds, and $\sum _{k \in \mathcal {K}_1 \cup \mathcal {K}_2}R_k(t)<\infty$, $t\ge 0$. Then, $\sum _{k_1 \in \mathcal {K}_1} \Pi _{k_1 }(t)$ and $\sum _{k_2 \in \mathcal {K}_2} \Pi _{k_2}(t)$ are independent Poisson processes, and $N(\mathcal {K}_1,\mathcal {K}_2)$ has a geometric distribution with success probability ${\sum _{k_2 \in \mathcal {K}_2} g_{k_2}}/\sum _{k \in \mathcal {K}_1 \cup \mathcal {K}_2}g_{k}$ which does not depend on $r(\cdot )$; that is,

2 $$\begin{aligned} \mathbb {P}\big \{ N(\mathcal {K}_1,\mathcal {K}_2) = n \big \} = \frac{\sum _{k_2 \in \mathcal {K}_2}g_{k_2}}{\sum _{k \in \mathcal {K}_1 \cup \mathcal {K}_2}g_{k}}\left( \frac{\sum _{k_1 \in \mathcal {K}_1} g_{k_1} }{\sum _{k \in \mathcal {K}_1 \cup \mathcal {K}_2}g_{k}} \right) ^{n} \hspace{5.69046pt}(n=0,1\ldots ). \end{aligned}$$

Moreover,

3 $$\begin{aligned} \frac{\sum _{k_2 \in \mathcal {K}_2} g_{k_2}}{\sum _{k \in \mathcal {K}_1 \cup \mathcal {K}_2} g_{k}} N(\mathcal {K}_1,\mathcal {K}_2) {\mathop {\longrightarrow }\limits ^{\mathcal {D}}} \ Exp(1) \ \text{ as } \frac{\sum _{k_2 \in \mathcal {K}_2} g_{k_2}}{\sum _{k \in \mathcal {K}_1 \cup \mathcal {K}_2 } g_{k}} \rightarrow 0. \end{aligned}$$

It immediately follows from (2) that $\mathbb {E} ( N(\mathcal {K}_1,\mathcal {K}_2) ) = \frac{\sum _{k_1 \in \mathcal {K}_1 }g_{k_1}}{\sum _{k_2 \in \mathcal {K}_2} g_{k_2}}$ and $\text{ Var }( N(\mathcal {K}_1,\mathcal {K}_2) ) = \left( \frac{\sum _{k_1 \in \mathcal {K}_1}g_{k_{1}}}{\sum _{k_2 \in \mathcal {K}_2}g_{k_{2}}} \right) ^{2}+\frac{\sum _{k_1 \in \mathcal {K}_1}g_{k_{1}}}{\sum _{k_2 \in \mathcal {K}_2}g_{k_{2}}}$. Thus, when Assumption (A2) holds, the mean number of type-$k_1$ founder B cells, $k_1\in \mathcal {K}_1$, joining the GC during the time interval $[0,\min _{k_2 \in \mathcal {K}_2} T_{k_21}]$ grows inversely proportional to the precursor frequency of type-$k_2$ cells, $k_2\in \mathcal {K}_2$: $\sum _{k_2 \in \mathcal {K}_2}g_{k_2}$.

#### Remark 2

(*Biological relevance*) The proportions $\{g_k, k\in \mathcal {K}\}$ represent the frequencies of antigen-specific B cells among each type of B-cell precursors recruited by GC. It has been estimated that, in humans, the frequency of VRC01-class CD4 binding site antibody precursors is about 1 in 2.4 million B cells. Precursors for other HIV-like bnAbs (e.g., PGT121) may be even more rare (Jardine et al. 2016; Steichen et al. 2016). The practical implication of the above results is that immunogens used by germline-targeting vaccines must substantially enrich the pool of on-target B-cells so they will not be outcompeted by off-target B cells selected for entry in GC induced by boost vaccination.

The continual seeding of GC has also implications on the diversification of the B-cell repertoire. Our results suggest that GC tend to be initially colonized by commonly found precursors (e.g., B cells founding clones with ‘average’ binding affinity potential, sufficient to enter GC) before they recruit the rarest ones (e.g., B cells able to produce high binding affinity B cells). By the time the first high affinity B cell joins the reaction, the GC will have been visited by many B cells with lower binding affinity potential. Each of these early founder B cells may initiate a clone at a time where competition for the antigen may not yet be at its peak. This mechanism may therefore support diversification of GC B cells during the earliest stage of the reaction. The waiting time until the highest binding affinity B cells join the reaction allows B cells with lower binding affinity to undergo somatic hypermutations, further diversifying the BCR repertoire. Thus, as a class, lower binding affinity B cells have a temporary competitive advantage over high binding affinity B cells due to the timing of their arrival which allows them to clonally expand. Although (rare) high binding affinity founder B cells tend to join GC later, their clones may still outnumber lower affinity clones if they have higher fitness. Another consequence of the continual seeding is clonal instability in the GC, a phenomenon studied in Sect. 5 which also diversifies the B-cell repertoire through the turnover of dominant clones.

### Branching mechanism and intra-clonal evolution

For every $k \in \mathcal {K}$, the lifespan of any type-*k* cell is described by a non-lattice r.v. $\tau _k$ with cumulative distribution function (c.d.f.) $G_k(x) = \mathbb {P}\{\tau _k \le x\}$, $x \in [0,\infty )$, with $G_k(0)=0$. At the end of its lifespan, every type-*k* cell produces a random number of offspring described by a r.v. $\xi _k = (\xi _{kj}, j \in \mathcal {K})$ where $\xi _{kj}$ denotes the number of type-*j* daughter cells. We assume that cells either die or divide; hence, $\sum _{j \in \mathcal {K}}\xi _{kj} \in \{0,2\}$. Let $h_k(\textbf{s}) = \mathbb {E}\left( \prod _{j=1}^K s_j^{\xi _{kj}}\right)$, $\textbf{s}=(s_1,\dots ,s_K)$, denote the probability generating function (p.g.f.) of $\xi _{k}$. Lastly, within a clone, every cell evolves independently of every other cell.

Let *C*(*k*) denote any type-*k* cell. Define $p_{0}^{k}=\mathbb {P}\{C(k)\rightarrow \emptyset \}$ and $p_{ij}^{k}=\mathbb {P}\{C(k)\rightarrow (C(i), C(j))\}$ where $p_{0}^{k}$ is the probability that any type-*k* cell completes its lifespan without producing any offspring either because it dies, or differentiates into a plasma or memory B cell, or exits the GC; and $p_{ij}^{k}$ denotes the probability that any type-*k* cell divides into two cells of types *i* and *j*, respectively, with $i,j,k\in \mathcal {K}$. Put $p_2^k = 1-p_0^k$ for the probability of division of any type-*k* cell. Let $q_{ij}^{k}=\mathbb {P}\{C(k)\rightarrow (C(i), C(j)) \vert C(k) \text{ divides } \} = {p_{ij}^{k}}/({1-p_0^k})$ denote the conditional probability that any type-*k* cell divides into one type-*i* and one type-*j* cell. The conditional probabilities $\{q^k_{ij}\}_{i,j\in \mathcal {K}}$ capture the impact of somatic hypermutation on type-*k* cells that manifests at division. Notice that $\{C(k)\rightarrow (C(i), C(j))\}=\{C(k)\rightarrow (C(j), C(i))\}$. Hence, for every $k \in \mathcal {K}$, the p.g.f. $h_k(\textbf{s})$ may be expressed as $h_{k}({\textbf{s}}) =p_{0}^{k}+\sum _{i\in \mathcal {K}} \sum _{j=i}^{K}p_{ij}^{k}s_{i}s_{j}$, $h_{k}(\textbf{1})=1.$

Let $m_{ij} = \mathbb {E}(\xi _{ij})=\frac{\partial h_{i}(\textbf{s})}{\partial s_{j}}\vert _{\mathbf {s=1}} = \sum _{n=1}^{j} p_{nj}^i + \sum _{n=j}^{K} p_{jn}^i$ denote the expected number of type-*j* cells produced by any type-*i* cell at the end of its lifespan. Define the associated matrix of mean offspring

$$\begin{aligned} M = \left( \begin{array}{cccc} m_{11}&{}\cdots &{}m_{1K}\\ \vdots &{}\ddots &{} \vdots \\ m_{K1}&{}\cdots &{}m_{KK} \end{array} \right) , \end{aligned}$$

and write $m_i = \sum _{j\in \mathcal {K}} m_{ij}$ for the total mean number of offspring produced by any type-*i* cell. Then, $m_{ij} = (1-p_0^i) \big ( \sum _{n=1}^{j} q_{nj}^i + \sum _{n=j}^{K} q_{jn}^i \big ).$ Let $\beta _{jk}^{i}=\frac{\partial ^{2}h_{i}(\textbf{s})}{\partial s_{j} \partial s_{k}}\vert _{\mathbf {s=1}}= \mathbb {E}\{\xi _{ij}(\xi _{ik}-\delta _{ik})\}$ where $\delta _{ik}=\textbf{1}_{\{i=k\}}$, for every $i,j,k \in \mathcal {K}$.

Let $\textbf{Z}(t) =(Z_{k}(t))_{k\in \mathcal {K}}$, $t\ge 0$, where $Z_k(t)$ is the number of type-*k* cells at time *t*, $k \in \mathcal {K}$, generated by the above-defined multitype branching process. Put $Z(t) = \sum _{k=1}^K Z_k(t)$ for the size of the clonal family at time *t*.

**Fig. 5:**
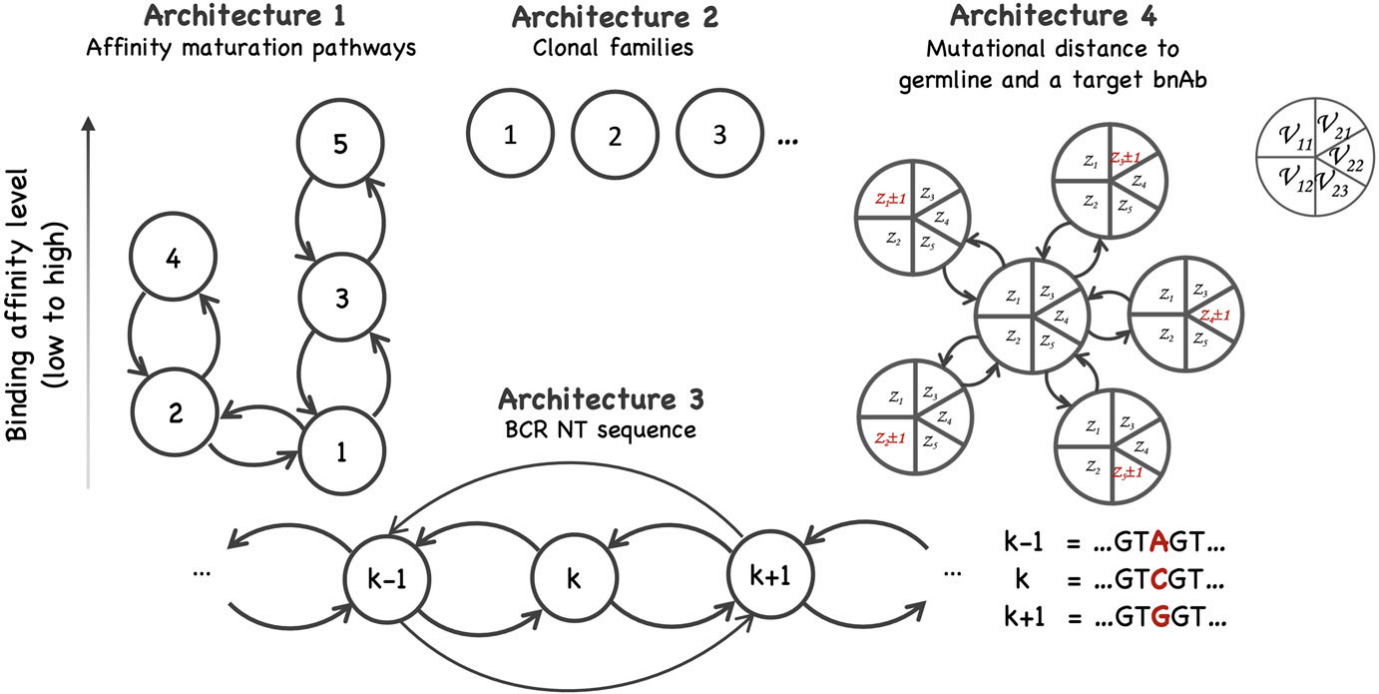
Four examples of network architectures describing the interconnection between cell types. In each example, the nodes represent the types and the edges identify the set of admissible transitions between types. For simplicity, self-renewing division (without change in type) and cell death are not shown in the graphs. *Architecture 1:* The process includes five types, with each type representing a binding affinity class (from lowest (1) to highest (5)) positioned along two mutational pathways; type-1 cells may produce type-2 and type-3 cells, and vice versa, allowing offspring to reversibly enter either one of the pathways: type-2 cells are less likely than type-3 cells to produce offspring of highest affinity (type-5); this first example is introduced in Sect. 3.5.1. *Architecture 2:* The model includes countably many types, each type representing one of the clonal families seeding a GC, ranked in the order in which they join the GC; in this second example, introduced in Sect. 3.5.2, the types do not connect. *Architecture 3:* Each type in the model represents the nucleotide sequence of the immunoglobulin gene loci that encode the heavy and/or light chains of the BCR; in this third example, briefly introduced in Sect. 3.5.3, transitions between types arise through somatic hypermutation. *Architecture 4:* The model offers a simplified description of the accumulation of somatic mutations in the Ig gene loci of B cells; the state space is a 5-dimensional vector: its first two entries represent the number of mutated and unmutated positions among those at which the germline and a target bnAb match ($\nu _{11}\cup \nu _{12}$); its last three entries represent the number of mutated and unmutated positions among those at which the germline and bnAb do not match ($\nu _{21}\cup \nu _{22}\cup \nu _{23}$); specifics about the model, in particular why three entries are needed in the latter case, are explained in Sect. 6

### Examples of model structures

We consider a few illustrative examples of model structures, shown in Fig. 5.

#### Example 1: modeling evolution of binding affinity classes

The role of GC is to generate high binding affinity B cells through somatic hypermutation, antigen-mediated selection, and clonal expansion. Our first model classifies the B cells that populate a GC into types that each represents a binding affinity class for one of the antigens fueling the reaction. The affinity level of an antibody refers to its strength in binding to a specific antigen and the stability of the resulting complex. A higher affinity indicates a stronger interaction between the antibody and antigen.

Many clonal families originate from a founding B cell with a relatively low affinity, but nevertheless sufficient to secure entry into the GC. Their descendants are subjected to somatic hypermutation which may either leave binding affinity virtually unchanged or altered. When their affinity improves, B cells are more likely to persist in the GC, captured by a decrease in the probability of death ($p_{0}^k$). The branching mechanism and topology of the transition network are formulated to describe propagation of the members of a clonal family over the various affinity classes under a given scenario. The example shown in Fig. 5 allows two mutational pathways. Both of them enable enhancement of affinity, but one is a mutational dead-end, prematurely restricting improvement of affinity. It extends the catenary model studied in Kleinstein and Singh (2001).

#### Example 2: an infinite-type model of clonal dominance

In the second example, GC B cells are classified according to the founder B cell from which they descend: descendants of the first founder are all of type 1; descendants of the second founder are all of type 2; and so on. Thus, here, each type identifies a particular clonal family, types do not communicate with each other, $\mathcal {K}=\{1,2\ldots \}$, and $K=\infty$. The offspring mean matrix is

4 $$\begin{aligned} M = 2 \left( \begin{array}{cccc} 1 - p_{0}^1&{}0&{} 0 &{} \cdots \\ 0 &{} 1 - p_{0}^2 &{} 0 &{} \cdots \\ \vdots &{}\ddots &{} \ddots &{} \end{array} \right) , \end{aligned}$$

and the lifespan of any type-*k* cell follows a distribution with c.d.f. $G_k(\cdot )$. We use this model to study the dynamics of clonal dominance within GC. The model predicts that GC are initially clonally highly diverse, before gradually loosing their clonal diversity and converging toward dominance by a few clonal families. These predictions corroborate previous experimental observations. Additionally, clones that dominate a GC reaction are eventually replaced by new ones, until the reaction stops.

#### Example 3: mutational gains and losses relative to a germline gene and a target antibody

Evolution of the BCR repertoire may be described at the sequence level by building a model in which each type represents the nucleotide sequence of a particular BCR and postulating how somatic hypermutations induce transitions between types at division (Fig. 5, architecture 3). However, the complexity of the rules of somatic hypermutation and affinity-based selection makes the formulation of such a model difficult (requiring specification of $\ge 4^L(4^L-1)$ transition probabilities for nucleotide sequences of length *L*). In this paper, we propose a simpler evolutionary model that focuses on gains and losses of mutations relative to a germline and a target antibody sequence. The model partitions positions of the heavy and/or light chain sequence into 5 distinct subsets to capture mutations at positions at which the bnAb and germline match or differ (Fig. 5, architecture 4; see also Sect. 6).

### Properties of the general model

Let $X_{\ell }$, $\ell =1,2\ldots$, be i.i.d. r.v. representing the type of the $\ell$-th founder B cell. When assumption (A1) holds, it follows from Fact 4 that $\mathbb {P} \big \{ X_{\ell } = k \big \} = {r_k(t)}/{r(t)}$, $k \in \mathcal {K}$. Let ${\textbf{Z}}^{(\ell )}(t)=\big ({Z}_{k}^{(\ell )}(t), k \in \mathcal {K} \big )$, $t\ge 0$, where $Z_k^{(\ell )}(t)$ denotes the number of type-*k* cells in the $\ell$-th clone *t* units of time after its initiation, and with initial condition ${\textbf{Z}}^{(\ell )}(0)= \textbf{e}_{X_{\ell }}$ where ${\textbf{e}}_k$ is a *K*-dimensional vector with all entries equal to 0 except for a one in the *k*-th place. We assume that:

(A3) $\{{\textbf{Z}}^{(\ell )}(\cdot )\}_{ \ell =1}^\infty$ are i.i.d. copies of $\mathbf{Z(\cdot )}$ started with one cell of type $\{X_\ell \}_{\ell = 1}^\infty$.

Put $\textbf{F}(t;\textbf{s})=\big (F_{k}(t;\textbf{s}), k \in \mathcal {K} \big )$ where $F_{k}(t;\textbf{s}) = \mathbb {E}\big (\textbf{s}^{\textbf{Z}^{(1)} (t)} \vert X_{1} = k \big )$ denotes the conditional p.g.f. of ${\textbf{Z}}^{(1)}(t)$, given the process begins with one type-*k* cell. These p.g.f. are the unique solutions of the nonlinear integral equations:

5 $$\begin{aligned} F_{k}(t;\textbf{s})=\int _{0}^{t} h_{k}(\textbf{F}(t-u;\textbf{s} ))dG_{k}(u)+s_{k}(1-G_{k}(t)) \hspace{14.22636pt}(k \in \mathcal {K}) \end{aligned}$$

with boundary conditions $F_{k}(0;\textbf{s})=s_{k}$, $k\in \mathcal {K}$ [see Mode (1971), Athreya and Ney (1972)].

For every $t\ge 0$, define $A_{kj}(t) = \mathbb {E} ( {Z}^{(1)}_j(t) \ \vert \ \textbf{Z}^{(1)}(0) = \textbf{e}_k )$ and $B_{ij}^{k}(t)=\mathbb {E} ( Z^{(1)}_{i}(t)(Z^{(1)}_{j}-\delta _{ij}) \vert {\textbf{Z}}^{(1)}(0) = \textbf{e}_k )$ where, for every $i,j,k \in \mathcal {K}$, $A_{kj}(t)$ denotes the average number of type-*j* cells at time *t* in a clone started from a single type-*k* founder B cell of age 0 at time 0, $B_{ij}^k(t)$ is the second-order factorial moment of the number of type-*i* and type-*j* cells at time *t* in a clone started from a single type-*k* cell at time 0, and $\delta _{ij}=1$ if $i=j$ and 0 otherwise. Put ${A}(t)=\left( A_{jk}(t), j,k \in \mathcal {K} \right)$. The variance of $Z_j(t)$ started from a single type-*k* cell is $V_{j}^{k}(t)=\text{ Var }(Z^{(1)}_{j}(t)\vert {\textbf{Z}}^{(1)}(0) = \textbf{e}_k)= B_{jj}^{k}(t)+A_{kj}(t)-A_{kj}^{2}(t)$. It follows from (5) that $A_{ij}(t)$ and $B_{jk}^{i}(t)$, $i,j,k \in \mathcal {K}$, satisfy the integral equations

6 $$\begin{aligned} A_{kj}(t)=\sum _{\ell =1}^{K}m_{k\ell }\int _{0}^{t}A_{\ell j}(t-u)dG_{k}(u)+\delta _{kj}(1-G_{k}(t)), \end{aligned}$$

and

7 $$\begin{aligned} B_{ij}^{k}(t)= & {} \sum _{\ell =1}^{K}m_{k\ell }\int _{0}^{t}B_{ij}^{\ell }(t-u)dG_{k}(u) \nonumber \\{} & {} +\sum _{k_1=1}^{K}\sum _{k_2=1}^{K}\beta _{k_1k_2}^{k}\int _{0}^{t}A_{k_1i}(t-u)A_{k_2 j}(t-u)dG_{k}(u). \end{aligned}$$

Let $G_k^*({s}) = \int _0^\infty e^{-s x} dG_k(x)$ denote the Laplace transform of $G_k(\cdot )$, $k\in \mathcal {K}$, and define the matrix

$$\begin{aligned} M^*( {s}) = \left( \begin{array}{cccc} G_1^*({s})m_{11}&{}\cdots &{}G_1^*({s})m_{1K}\\ \vdots &{}\ddots &{} \vdots \\ G_K^*({s})m_{K1}&{}\cdots &{}G_K^*({s})m_{KK} \end{array} \right) . \end{aligned}$$

If $M^*( {s})$ is irreducible, then its Perron-Frobenius root $\rho ^*(s)$ always exists, and we can define [see Mode (1971)]:

#### Definition 1

(*Malthusian parameter*) The Malthusian parameter $\alpha$ is the solution, assuming it exists, of the equation $\rho ^*(\alpha ) = 1$ where $\rho ^*(\alpha )$ denotes the Perron–Frobenius root of $M^*({\alpha })$ (i.e., $\rho ^*(\alpha )$ is the largest eigenvalue of $M^*(\alpha )$). The Malthusian parameter $\alpha$ always exists in the super- and critical cases; its existence must be verified in the subcritical case. When $\alpha$ exists, we have $\alpha <0$, $=0$, or $>0$ depending on whether the process is subcritical, critical, or supercritical, respectively.

Suppose that *M* is positive regular (i.e., $\exists n \in \{1,2\ldots \}$ such that the entries of ${M}^n$ are all strictly positive), and the p.d.f. $g_k(t)= dG_k(t)/dt$, $k\in \mathcal {K}$, are squared integrable. Let ${\textbf{u}} = (u_k, k \in \mathcal {K})$ and ${\textbf{v}} = (v_k, k \in \mathcal {K})$ denote strictly positive right and left eigenvectors of ${M}^*(\alpha )$ associated with its Perron–Frobenius root and chosen so that ${\textbf{u}} \textbf{1}^\top = {\textbf{u}}{\textbf{v}}^\top = 1$ where $\textbf{1}= (1,\dots ,1)$. Then, the average intra-clonal composition, normalized by $e^{\alpha t}$, stabilizes over time: $e^{-\alpha t} A(t) \rightarrow C$, as $t\rightarrow \infty$, for some fixed matrix $C = (c_{jk}, j,k \in \mathcal {K})$ (Mode 1971). When $K > 1$ (finite), this result remains valid under mild assumptions. When $K=\infty$, it may still hold depending on the offspring and lifespan distributions.

Let $\textbf{W}(t)=e^{-\alpha t} \textbf{Z}(t)$. We may assume that

(A4) In the supercritical case ($\alpha >0$), $\textbf{W}(t){\mathop {\longrightarrow }\limits ^{a.s.}} W \textbf{v}$ as $t\rightarrow \infty$ for some r.v. *W* such that $W=0$ (or $>0$) a.s. if $\lim \limits _{t\rightarrow \infty }{} \textbf{Z}(t)=\textbf{0}$ (or $\ne \textbf{0}$).

#### Remark 3

For single-type Bellman–Harris processes, specific conditions under which Assumption (A4) holds are given in Harris (1963) (Ch. VI, Theorem 21.1 and Corollary). For multitype Markov branching processes, see Athreya and Ney (1972) (Ch. V, Sect. 7.5, Theorem 2). For multitype age-dependent branching processes, see Mode (1971) (Sect. 3.13, Theorem 13.1). Also, convergence may hold in probability or in mean square instead of almost surely.

#### Remark 4

Intra-clonal dynamics is affected by whether the clonal family ultimately survives. Assuming (A4) holds, and letting $q = \mathbb {P}\{W=0\}$ denote the probability of extinction, we have conditional on non-extinction that

8 $$\begin{aligned} \mathbb {P}\left\{ e^{-\alpha t} \textbf{Z}(t) \le \textbf{x} |{Z}(t)>0 \right\} {\mathop {\longrightarrow }\limits ^{t\rightarrow \infty }} 1 - \frac{1-\mathbb {P}\{ W\textbf{v} \le \textbf{x} \}}{1-q}. \end{aligned}$$

Otherwise, the process converges to 0 as $t\rightarrow \infty$ (extinction).

To describe the dynamics of GC B cells, define the vector $\textbf{Y}(t)= \left( {Y}_k(t), k \in \mathcal {K} \right)$, $t\ge 0$, where $Y_k(t)$ denotes the number of type-*k* B cells at time *t* in the GC. We assume that the GC does not contain any B cell at time $t=0$, and set $\textbf{Y}(0)= \textbf{0}$ where $\textbf{0}$ denotes the *K*-dimensional null vector. Define $Y(t):= \sum _{k=1}^K Y_k(t)$. For every $t\ge 0$, $\textbf{Y}(t)$ can be expressed as

9 $$\begin{aligned} \textbf{Y}(t)=\sum _{\ell =1}^{\Pi (t)}{\textbf{Z}}^{(\ell )}(t-T_{\ell })\textbf{1}_{\{\Pi (t)>0\}}, \end{aligned}$$

where ${\textbf{Z}}^{(\ell )}(u)= \big ( {Z}_k^{(\ell )}(u) \big )_{ k \in \mathcal {K}}$ represents the composition of the $\ell$-th clone *u* units of time after it started, setting ${\textbf{Z}}^{(\ell )}(u)=0$, $u<0$. We refer to $\textbf{Y}(\cdot )$ as a *K*-type age-dependent branching process with non-homogeneous immigration.

Let $\Phi (t;\textbf{s})=\mathbb {E}\{\textbf{s}^{\textbf{Y} (t)}\vert \textbf{Y}(0)=\textbf{0}\}$ denote its p.g.f., $\textbf{M}(t) = \big ( M_i(t) \big )_{i \in \mathcal {K}}$ and $\textbf{C}(t) = \big ( C_{ij}(t) \big )_{i,j \in \mathcal {K}}$ where $M_i (t)=\mathbb {E}(Y_i(t) \vert \textbf{Y}(0)=\textbf{0})$ and $C_{ij}(t) = \text{ Cov }(Y_{i}(t),Y_{j}(t)\vert \textbf{Y}(0)=\textbf{0})$ denote the expectation and covariance of $\textbf{Y}(t)$. Adapting a result from Mitov et al. (2018), we deduce when Assumptions (A1,A2) hold that

10 $$\begin{aligned} \Phi (t;\textbf{s})=\exp \left( -\int _{0}^{t}r(t-x)\left[ 1-\sum _{i\in \mathcal {K}} g_i {F}_i(x;\textbf{s}) \right] dx\right) , \end{aligned}$$

with the boundary condition $\Phi (0;\textbf{s})=\textbf{1}$. Moreover, for every $j,k\in \mathcal {K}$,

11 $$\begin{aligned} M_j (t) = \int _0^t r(t-x) \overline{A}_{j}(x) dx \quad \text{ and } \quad C_{j k } (t) = \int _0^t r(t-x) \overline{C}_{j k}(x)dx, \end{aligned}$$

where $$\overline{A}_{j}(x)=\sum _{i \in \mathcal {K}} g_i A_{ij}(x)$$ and $$\overline{C}_{jk}(x)=\sum _{i\in \mathcal {K}} g_{i}B_{jk}^{i}(x)$$.

### Classes of B cells that communicate do not vanish

**Fig. 6:**
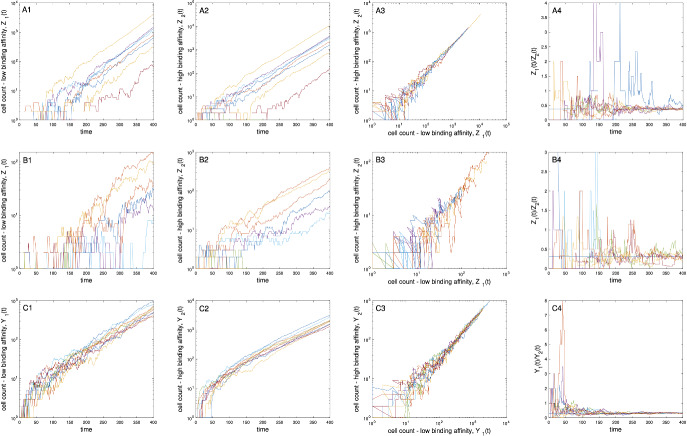
*Behavior of a two-type process, with and without immigration.* Panels A1-A4 show results from 10 independent simulations of the model without immigration, with $(m_{11},m_{12},m_{21},m_{22})=(0.20,0.50,0.55,0.20)$ and $G(\cdot )$ assumed exponential with parameter $\lambda = 1/24$. Each trajectory represents the evolution of one clone. Each of them begins with one type-2 ($\sim$ high binding affinity) cell. Panel A1 (resp., A2): number of low (resp., high) binding affinity B cells per clone over time; Panel A3: count of high versus low binding affinity B cells which grow at the same rate; Panel A4: ratio of the numbers of low and high binding affinity B cells plotted over time; the black straight line indicates its a.s. limit $\simeq 0.37$. Panels B1-B4 are identical to panels A1-A4, except that simulations were conducted by setting $(m_{11},m_{12},m_{21},m_{22})=(0,0.30,0.55,0.20)$. The a.s. limit is $\simeq 0.31$ in this setting. Panels C1-C4 are similar to those above, but the model now includes immigration. The parameters of the branching process were identical to those used for panels B1-B4; the immigration Poisson process was time-homogeneous and assumed that $g_1 = g_2 = 0.5$ and $r_0=0.25$

Under Assumption (A4), the proportion of type-*k* cells in a clonal family not going extinct tends a.s. to $v_{k}/\sum _{k'=1}^K v_{k'}$ as $t\rightarrow \infty$. Since $\textbf{v}$ has strictly positive entries, all types are predicted to grow at the same exponential rate $\alpha$, regardless of the probability at which they die or self-renew. This property arises from the irreducibility of the mean offspring matrix *M* (i.e., the fact that all types communicate). It is illustrated in Fig. 6 using simulations based on a two-type process. From a biological standpoint, communication between types is driven by somatic hypermutation. Its biological interpretation in the context of Model 1 (Sect. 3.5.1) is that clonal families in which high binding affinity B cells can mutate into lower binding affinity B cells (and vice versa) may include non-negligible numbers of B cells of varying binding affinity levels, even those less likely to survive. Kuraoka et al. (2016) made a similar observation in mouse experiments, reporting clonally related B cells competing for a same epitope but with affinity orders of magnitude different (Kuraoka et al. 2016). They observed cases where low-affinity B cells accounted for 22–27% of GC B cells. This observation, which has been interpreted as an indication of the permissiveness of GC to retain low or moderate affinity B cells (Kuraoka et al. 2016; Bannard and Cyster 2017), is not fully understood. While several factors could explain it, including the possibility of an affinity threshold (Kuraoka et al. 2016), the asymptotic behavior of $\textbf{Z}(t)$ indicates that clonal expansion and somatic hypermutation could also jointly contribute.

### On selection and competition between B cells

Several processes contribute to the regulation of B cells in GC. Homeostasis maintains a balance between B-cell amplification and excessive immune activation. Additionally, antigen-mediated selection favors B cells that have undergone mutations that result in higher affinity for the antigen. These selected B cells receive stronger survival signals compared to those with lower affinity, which are more prone to undergoing apoptosis.

The model accounts for some, but not all, of these processes. For instance, it captures, at least partially, variations in antigen-mediated selection among B cells by introducing type-specific probabilities of cell death ($p_k^0$). These probabilities can differ, resulting in some cell types in the model having lower fitness and being more susceptible to cell death than others.

However, the model does not consider the homeostatic mechanisms that regulate the growth of GC B-cell populations. Specifically, in cases where a clonal family is driven by a supercritical process that does not go extinct, its size will grow exponentially. Therefore, when describing the composition of a population based on cell counts, the model is best suited for depicting the organization of GC or individual clonal families during their early phase. Over extended time periods, the model can be used to track the temporal evolution of the relative frequencies of B cell classes using $\textbf{Y}(t)/Y(t)$ or $\textbf{Z}(t)/Z(t)$.

Another critical aspect of GC dynamics involves the competition among B cells, both between and within clonal families and types. When a new B cell with higher affinity than those already present in the GC joins the population, competition could increase the likelihood of death of other, less competitive cell types, resulting in a reduction of their Malthusian parameter. While the model does not explicitly detail this phenomenon, it partially captures it when comparing the growth of two distinct cell types. To illustrate, consider two different, non-communicating cell types $k\ne k'$. Assume that each of them satisfies Assumption (A4) such that $Z_k(t-T_k) \sim v_k W_k e^{\alpha _k (t-T_k)}$ and $Z_{k'}(t-T_{k'}) \sim v_{k'} W_{k'} e^{\alpha _{k'} (t-T_{k'})}$. The ratio $Z_k(t)/Z_{k'}(t)$, representing the odds of selecting a cell of type *k* versus type $k'$, provides insights into how the frequencies of cell types *k* and $k'$ evolve over time. We use it in Sect. 5 to compare the size of clonal families. For every $t\ge \max (T_k,T_{k'})$, conditional on non-extinction for both clonal families ($W_kW_{k'}>0$), this ratio satisfies:

$$\begin{aligned} \frac{Z_k(t-T_k)}{Z_{k'}(t-T_{k'})} {\mathop {\sim }\limits ^{a.s.}} \frac{W_kv_k e^{-\alpha _{k}T_k} }{W_{k'}v_{k'} e^{-\alpha _{k'}T_{k'}}} e^{(\alpha _{k}-\alpha _{k'})t} \quad (\text{ as } t\rightarrow \infty ). \end{aligned}$$

Thus, the odds would remain similar to those in a model where the Malthusian parameter in the *k*-th clone decreases upon the initiation of the more competitive $k'$-th clonal family, provided that the difference between the Malthusian parameters is $\alpha _{k'} - \alpha _k$. Overall, despite some limitations, we have observed that the model can effectively replicate certain experimental observations.

## Temporal diversity of the B-cell repertoire

A diverse BCR repertoire is essential for effective immune protection against infection and a potential benefit for responses to antigens structurally related to those from past infections or vaccinations (Kuraoka et al. 2016). However, it can also give rise to significant amounts of B cells expressing binding antibodies unlikely to progress toward pathogen neutralization; instead, these B cells may compete with and restrict those capable of generating neutralizing antibodies. We propose to measure evolution of diversity over time in terms of the *K* types of the model to gain further insights into the organization of the B-cell repertoire. In this regard, we introduce indices of temporal $\alpha$ and $\beta$ diversities for multitype branching processes. We applied them in Sect. 5 to study the turnover of clonal families within GC.

### Temporal alpha diversity

Alpha diversity is a fundamental concept in ecology to quantify species diversity within a given habitat. Popular alpha diversity indices include the Hill numbers. When applied to the proposed multitype branching process with immigration to quantify the diversity of types in a GC at time *t*, the Hill number of order *q*, defined on the event $\{Y(t) = \sum _{k=1}^K Y_k(t)>0\}$, is given by

12 $$\begin{aligned} D_Y^{q}(t) = \left( \sum \limits _{k=1}^K \frac{Y_k(t)^q}{Y(t)^q} \right) ^{1/(1-q)} \in [1,K^{1/(1-q)}], \end{aligned}$$

where $q \in (0,1)\cup (1,\infty )$ determines the sensitivity of the index to the relative frequency of each type, with the influence of the most abundant types increasing with *q* (Hill 1973). The higher $D_Y^{q}(t)$, the higher diversity. Letting $q\rightarrow 0$, $D_Y^{q}(t)$ tends to $D_Y^{0}(t)=\sum _{k=1}^K \textbf{1}_{\{Y_k(t)>0\}}$ (species richness), representing the total number of types in the GC at time *t*. Setting $q=2$ yields the inverse Simpson diversity index (Simpson 1949) used in Sect. 5 to assess evolution of clonal diversity, whereas letting $q\rightarrow 1$ gives the exponential of the Shannon index:

13 $$\begin{aligned} D_Y^{1}(t):= \exp \left( - \sum \limits _{k=1}^K \frac{Y_k(t)}{Y(t)} \log \left( \frac{Y_k(t)}{Y(t)}\right) \right) \in [1,K]. \end{aligned}$$

Clone-specific Hill numbers are defined on $\{Z(t) = \Vert \textbf{Z}(t)\Vert >0\}$ as $D_Z^{q}(t) = \left( \sum \nolimits _{k=1}^K \frac{Z_k(t)^q}{Z(t)^q} \right) ^{1/(1-q)}$. Since the summands in $D_Y^{q}(t)$ and $D_Z^{q}(t)$ are positive, both $D_Y^{q}(t)$ and $D_Z^{q}(t)$ are well defined when $K=\infty$.

#### Proposition 1

Suppose that Assumption (A4) holds. Then, conditional on non-extinction ($W>0$), we have for every $K\in \{1,2\ldots \}$ that $\lim \nolimits _{t\rightarrow \infty } D_Z^{q}(t) {\mathop {=}\limits ^{a.s.}} \left( { \sum \nolimits _{k=1}^K v_k^q}/{(\sum _{k'=1}^K v_{k'})^q} \right) ^{1/(1-q)}$ for every $q\in (0,1)\cup (1,\infty )$, $\lim \nolimits _{t\rightarrow \infty } D_Z^{q}(t) {\mathop {=}\limits ^{a.s.}}K$ as $q\rightarrow 0$, and $\lim \nolimits _{t\rightarrow \infty } D_Z^{1}(t) {\mathop {=}\limits ^{a.s.}} \exp \left( - \sum \nolimits _{k=1}^K \frac{v_k}{\sum \nolimits _{k=1}^K v_k} \log \left( \frac{v_k}{\sum \nolimits _{k=1}^K v_k}\right) \right)$ as $q\rightarrow 1$.

The proof follows easily from Assumption (A4) and Proposition 1 in Hill (1973).

### Temporal beta diversity

The variation in species composition between two habitats or time points may be measured using beta diversity indices (Whittaker 1960, 1972). We consider these indices to quantify the degree of temporal dissimilarity (turnover) of the *K* types in a clonal family and a GC. These indices are defined using either presence-absence, or abundances (counts), or relative abundances (proportions) of types. The Jaccard distance (Jaccard 1912) between the collection of types present in a clonal family at times $t_1$ and $t_2$ is defined using absence-presence of types as

14 $$\begin{aligned} J_Z(t_1,t_2) = 1-\frac{|\mathcal {S}(t_1) \cap \mathcal {S}(t_2) |}{|\mathcal {S}(t_1) \cup \mathcal {S}(t_2) |} \in [0,1], \end{aligned}$$

where $\mathcal {S}(t)=\{k\in \mathcal {K}:Z_k(t)>0\}$ is the set of types in the clone at time *t*. A value of 0 indicates identical type representation at times $t_1$ and $t_2$, and 1 no overlap in types. This index does not take frequency of types into account in measuring diversity. The Bray–Curtis index, which quantifies the degree of compositional dissimilarity within a clonal family at times $t_1$ and $t_2$ using

15 $$\begin{aligned} BC_Z(t_1,t_2) = \sum _{k=1}^K \frac{ |Z_{k}(t_1)-Z_k(t_2)|}{Z(t_1)+Z(t_2)} \in [0,1], \end{aligned}$$

is defined based on the vector of counts $\textbf{Z}(t)$ and normalized by $Z(t_1)+Z(t_2)$ to range between 0 (no compositional variation) and 1 (full turnover of types). A popular beta diversity index based on relative abundances to measure the overlap between the composition of a clonal family at times $t_1$ and $t_2$ is the Hellinger distance $H_Z(t_1,t_2) = \sqrt{1 - B_Z(t_1,t_2)}$ where

16 $$\begin{aligned} B_Z(t_1,t_2) = \sum _{k=1}^K \sqrt{\frac{Z_{k}(t_1)}{Z(t_1)}} \sqrt{\frac{Z_{k}(t_2)}{Z(t_2)}}\in [0,1] \end{aligned}$$

is the Bhattacharyya coefficient, representing the cosine of the angle between the vectors $\sqrt{ \textbf{Z}(t_1)/Z(t_1)}$ and $\sqrt{\textbf{Z}(t_2)/Z(t_2)}$ (Bhattacharyya 1946). Another similar index also based on the relative abundances $\textbf{Z}(t)/Z(t)$ is the proportion of similarity (Whittaker 1972):

17 $$\begin{aligned} P_Z(t_1,t_2) = \sum _{k=1}^K \min \left\{ {\frac{Z_{k}(t_1)}{Z(t_1)}}, {\frac{Z_{k}(t_2)}{Z(t_2)}} \right\} = 1 - \frac{1}{2}\sum _{k=1}^K \left|\frac{Z_{k}(t_1)}{Z(t_1)} - \frac{Z_{k}(t_2)}{Z(t_2)} \right|. \end{aligned}$$

Both $B_Z(t_1,t_2)$ and $P_Z(t_1,t_2)$ range between 0 (no compositional similarity between $t_1$ and $t_2$) and 1 (perfect similarity). The Hellinger distance $H_Z(t_1,t_2)$ also ranges between 0 (absence of change in the proportion of types from $t_1$ to $t_2$) and 1 (complete turnover of types). These indices are all defined conditional on $Z(\max (t_1,t_2))>0$. They emphasize different aspects of the composition of a clonal family. For multitype branching processes, they behave as follows:

#### Proposition 2

Let $K\in \{1,2\ldots \}\cup \{\infty \}$, $\delta \ge 0$, and suppose that Assumption (A4) holds. Then, conditional on $\{W>0\}$, we have as $t\rightarrow \infty$ that $J_Z(t,t+\delta ){\mathop {\longrightarrow }\limits ^{a.s.}} 0$,

$$\begin{aligned} BC_Z(t,t+\delta ) {\mathop {\longrightarrow }\limits ^{a.s.}} bc_Z(\delta ,\alpha ):= \frac{1-e^{-\alpha \delta }}{1+e^{-\alpha \delta }} \sim 1-e^{-\alpha \delta } \quad \text{ as } \alpha \delta \rightarrow \infty , \end{aligned}$$

whereas $H_Z(t,t+\delta ){\mathop {\longrightarrow }\limits ^{a.s.}} 0$, $B_Z(t,t+\delta ){\mathop {\longrightarrow }\limits ^{a.s.}} 1$, and $P_Z(t,t+\delta ){\mathop {\longrightarrow }\limits ^{a.s.}} 1$, for every $\delta >0$.

See Sect. 7.1 for a proof. Proposition 2 shows that the Bray–Curtis index of the compositional beta diversity of a clonal family not becoming extinct converges over time to a finite constant in the admissible range [0, 1], depending on $\delta$. Convergence to 1 as $\delta \rightarrow \infty$ occurs exponentially fast with $\delta$ and reflects the continual expansion of the clonal family as $\delta$ increases. Interestingly, asides from $\delta$, the limit in *t* depends solely on $\alpha$. Thus, as $t\rightarrow \infty$, the Bray–Curtis index becomes insensitive to any aspect of the offspring and lifespan distributions beyond those captured by the Malthusian parameter, and to the number of types, *K*. It partitions the family of supercritical multitype branching processes into equivalence classes indexed by $\alpha$ and including processes with identical Bray–Curtis diversity. Hence, two non-extinct multitype branching populations with identical Bray–Curtis indices have similar Malthusian parameter $\alpha$, whereas when their Bray–Curtis indices differ, the one with the highest index is expected to have a larger Malthusian parameter because $bc_Z(\delta ,\alpha )$ increases with $\alpha$. It follows easily from Proposition 2 that

$$\begin{aligned} \hat{\alpha }_Z(t,\delta ) = \frac{1}{\delta } \log \left( \frac{1+BC_Z(t,t+\delta )}{1-BC_Z(t,t+\delta )} \right) \quad {\mathop {\longrightarrow }\limits ^{a.s.}}\alpha \quad \text{ as } t\rightarrow \infty \end{aligned}$$

for every $\delta \ge 0$ if Assumption (A4) holds and conditional on non-extinction.

The indices $H_Z(t,t+\delta )$, $B_Z(t,t+\delta )$, and $P_Z(t,t+\delta )$ tend to 0 or 1 as $t\rightarrow 0$ regardless of $\delta$. The behavior of these indices reflects intra-clonal stabilization of the frequency of the types as *t* increases. See (Jagers and Nerman 1996) for further discussion on the asymptotic composition of supercritical multitype branching processes.

Indices of beta diversity may also be defined at the GC level; for example,

18 $$\begin{aligned} BC_Y(t_1,t_2) = \sum _{k=1}^K \frac{ |Y_{k}(t_1)-Y_k(t_2)|}{Y(t_1)+Y(t_2)}, \quad B_Y(t_1,t_2) = \sum _{k=1}^K \sqrt{\frac{Y_{k}(t_1)}{Y(t_1)}} \sqrt{\frac{Y_{k}(t_2)}{Y(t_2)}}, \end{aligned}$$

and $H_Y(t_1,t_2) = \sqrt{1 - B_Y(t_1,t_2)}$. The asymptotic behavior of GC-specific indices must be treated on a case-by-case basis. For the model studied in Sect. 5, $H_Y(t,t+\delta )$ converges a.s. to 0 as $\delta \rightarrow \infty$ when the branching mechanism is the same across clonal families.

## Oligoclonality, diversity of clonal families, and instability of clonal dominance

### Background and notation

Germinal center reactions begin polyclonal and tend to finish oligoclonal (Kroese et al. 1987; Küppers et al. 1993; Faro and Or-Guil 2013; Tas et al. 2016). The cross-sectional experiments that established these findings did not capture the temporal trajectory of individual clones. Thus, for example, we do not know whether clones that dominate a GC remain dominant over time.

To gain further insights into the dynamics of clonal dominance, we consider the infinite-type process introduced in Sect. 3.5.2 and formulate the overall immigration process $\Pi (\cdot )$ as a time-inhomogeneous Poisson process with rate $r(\cdot )$. The order of arrival induced by this process determines the types in the model. Since each type represents a specific clone, the sequence $\{T_{k\ell }\}_{k=1}^\infty$ a.s. satisfies $T_{k1}=T_k$ and $T_{k \ell }=\infty$, $\ell =2,3\ldots$. The first identity indicates that the time at which the first member of the *k*-th clone joins the GC ($T_k$) is the time at which the first type-*k* founder B cell joins the GC. The second identity ensures that each clone has a unique arrival time in the GC. Consequently, the type-specific immigration processes $\{\Pi _k(\cdot )\}_{k=1}^\infty$ are neither Poisson processes nor independent. The composition of the *k*-th clone at time *t* is ${\textbf{Z}^{(k)}(t)}=\big (0,\dots ,0, Z^{(k)}_k(t),0,\dots \big )$; hence, $\textbf{Y}(t)=\big (Z^{(1)}_1(t-T_1),Z^{(2)}_2(t-T_2),\dots \big )$ from (9).

For every $k\in \mathcal {K}=\{1,2\ldots \}$, the lifespan of every type-*k* B cell in the GC is a r.v. with c.d.f. $G_k(\cdot )$, and their probability of division is $p_2^k$. Since binding affinity may differ between clones, we assume that these probabilities are independent and identically distributed (i.i.d.) r.v. with c.d.f. $H(x) = \mathbb {P}\{p_{2}^k \le x \}$, $x\in [0,1]$. Likewise, the c.d.f. $G_k(\cdot )$ could be assumed to be randomly sampled from a family of distribution functions. Here, none of the types communicate and, conditional on $\{p_2^k,G_k(\cdot )\}$, we define the Malthusian parameter of the *k*-th clone as the root $\alpha _k$ of the equation

19 $$\begin{aligned} 2p_{2}^k \int _0^\infty e^{- \alpha _k x} dG_k(x) = 1, \end{aligned}$$

assuming such a root exists a.s.; it always does when $p_{2}^k \ge 0.5$. When each $G_k(\cdot )$ is an exponential distribution with parameter $\lambda$, the Malthusian parameters are given by $\alpha _k = (2 p_2^k-1)\lambda$ and form a sequence of i.i.d. r.v. with c.d.f.

20 $$\begin{aligned} \mathbb {P}\{\alpha _k \le u\} = \mathbb {P}\{p_2^k\le (1+u/\lambda )/2\} = H \left( \frac{\lambda +u}{2 \lambda } \right) \hspace{5.69046pt}(u\in (-\lambda ,\lambda )). \end{aligned}$$

The conditional probability of extinction of the *k*-th clone, given $\{p_2^k,G_k(\cdot )\}$, defined as the smallest non-negative root of the equation $f_k(s)=s$ where $f_k(s)=1-p_2^k+p_2^ks^2$, is $p_{ext}^k = \min \{1,{p_2^k}/(1-{p_2^k})\}$. If Assumption (A4) holds for the *k*-th clone, it follows that $Z^{(k)}_k(t){\mathop {=}\limits ^{a.s.}}c_k'W_ke^{\alpha _k t}(1+o(1))$ where $c_k'=(m_k - 1)/\alpha _k m_k^2\int _0^\infty ue^{-\alpha u}dG_k(u)$ and $m_k=2p_k^2$ (Athreya and Ney 1972).

#### Definition 2

(*Clonal dominance*) We say that the *k*-th clone dominates the GC reaction at time *t* if its size is equal to or exceeds that of any other clone at time *t*; that is, if $Z^{(k)}_k(t-T_k) \ge Z^{(k')}_{k'}(t-T_{k'})$, $k'\in \{1,\ldots ,\Pi (t)\} {\setminus }\{k\}$.

We study temporal evolution of clonal dominance using $F_k(t)=Z_k^{(k)}(t-T_k)/Y(t)$, the fraction of B cells in the GC at time *t* belonging to the *k*-th clone. We set $F_k(t)=0$ when $Y(t)=0$. We study evolution of the alpha and beta diversities of clonal families within GC using indices introduced in Sect. 4. Following initiation of a GC, species (clonal) richness is given by

$$\begin{aligned} D^0_Y(t)=\sum _{k=1}^{\Pi (t)} \textbf{1}_{\{Z_k^{(k)}(t-T_k)>0\}} \in [0,\Pi (t)]\quad (t\ge 0). \end{aligned}$$

Assuming independence of $\Pi (t)$ and $\{Z_k^{(k)}(t-T_k)\}_{k=1}^\infty$, the expected count of clonal families satisfies $\mathbb {E}(D^0_Y(t))\in [(1-Q)R(t),R(t)]$ where $Q = \lim \limits _{t\rightarrow \infty }\mathbb {P}\{Z_1^{(1)}(t)=0\}$. Species richness does not account for the abundance of non-extinct clonal families and grows indefinitely with *t* when $Q>0$. In particular, when $r(t)=r_0$, $t\ge 0$, it can be shown that $\mathbb {E}(D^0_Y(t))\sim r_0(1- Q) t$, such that increasing in the probability of extinction of clonal families (and of cell death) will proportionally reduce the number of clonal families in the GC. The Simpson index (Simpson 1949) which accounts for the abundance of clonal families and specializes to

21 $$\begin{aligned} S_Y(t):= \sum _{k=1}^{\Pi (t)} \frac{Z_k^{(k)}(t-T_k)^2}{Y(t)^2} = \sum _{k=1}^{\Pi (t)} F_k(t)^2 \in [0,1], \end{aligned}$$

is the probability that two cells sampled with replacement at time *t* from a given GC belong to a same clonal family. The inverse of the Simpson index $D_Y^2(t) = S_Y(t)^{-1}$ provides a measure of the alpha clonal diversity: the higher $S(t)^{-1}$, the larger the clonal diversity, accounting for evenness (Rempala and Seweryn 2013). The Bray–Curtis index and proportion of similarity comparing clonal composition in a GC at times $t_1$ and $t_2$ ($t_1\ge t_2$) are $BC_Y(t_1,t_2) =\sum _{k=1}^{\Pi (t_2)} \frac{\vert Z_k^{(k)}(t_1-T_k)-Z_k^{(k)}(t_2-T_k)\vert }{Y(t_1)+Y(t_2)}$ and $P_Y(t_1,t_2) = 1 - \frac{1}{2} \sum _{k=1}^{\Pi (t_2)} \big \vert \frac{ Z_k^{(k)}(t_1-T_k)}{Y(t_1)} - \frac{ Z_k^{(k)}(t_2-T_k) }{Y(t_2)}\big \vert .$ The Bhattacharyya coefficient is $B_Y(t_1,t_2) = \sum _{k=1}^{\Pi (t_2)} \sqrt{F_k(t_1)F_k(t_2)}.$ Here, we ask what these indices reveal about clonal dynamics in the absence and presence of differential fitness between clones, as previously considered (Tas et al. 2016). We note that the clone-specific diversity indices are $D_Z^2(t)=0$, $S_Z(t)=1$, and $B_Z(t,t+\delta ) =0$ for every $t, \delta \ge 0$, because each clone includes a single type.

### Clonal dynamics in the absence of differential fitness

In this section, we assume absence of differential fitness between clones.

#### Proposition 3

Suppose that all clones have identical offspring and lifespan distributions. Let $\alpha$ denote their common Malthusian parameter. Suppose that $\alpha >0$, Assumption (A4) holds, and (A5) there exists a positive, increasing function $f(\cdot )$ such that $f(t)=o(t)$ and $\lim _{t\rightarrow \infty }R(t-f(t))/R(t)=1$. Then, for every $\delta \ge 0$, as $t\rightarrow \infty$, $F_k(t) {\mathop {\longrightarrow }\limits ^{a.s.}} {{ W_k e^{- \alpha T_k}}}/{{\sum _{k'=1}^{\infty } W_{k'} e^{-\alpha T_{k'}}}}$, $k = 1,2\ldots$, $S_Y(t) {\mathop {\longrightarrow }\limits ^{a.s.}} {\sum _{k=1}^\infty W_k^2e^{-2\alpha T_k}}/{(\sum _{k'=1}^\infty W_{k'}e^{-\alpha T_{k'}})^2}\in (0,1)$, $J_Y(t,t+\delta ) {\mathop {\longrightarrow }\limits ^{\mathbb {P}}} 0, \ BC_Y(t,t+\delta ) {\mathop {\longrightarrow }\limits ^{a.s.}} ({1-e^{-\alpha \delta }})/({1+e^{-\alpha \delta }}),\ H_Y(t,t+\delta ) {\mathop {\longrightarrow }\limits ^{a.s.}} 0$, $B_Y(t,t+\delta ){\mathop {\longrightarrow }\limits ^{a.s.}} 1$, and $P_Y(t,t+\delta ){\mathop {\longrightarrow }\limits ^{a.s.}} 1.$

See Sect. 7.2 for a proof. Proposition 3 shows that, in the absence of differential fitness between clones, clonal dominance is a stable property unlikely to be challenged by other clones once established. Since the relative abundance $F_k(t)$ is asymptotically proportional to $e^{- \alpha T_k}{\mathop {\longrightarrow }\limits ^{a.s.}} 0$ ($k\rightarrow \infty$) and $\{W_k\}_{k=1}^\infty$ is an i.i.d. sequence, clones that arrive early in the GC are the most likely to become dominant. The relative size of the dominant clone tends to increase with $\alpha$ and decrease with the immigration rate (causing $T_k-T_{k-1}$ to stochastically decrease). Although the smaller $\alpha$, the longer it may take for clonal dominance to establish, the replacement of a dominant clone by another clone joining the reaction later is unlikely to happen because this would require a sequence of independent r.v. with a decreasing trend to break a record value that increases with *k*: suppose the $k^*$-th clone is dominant and $W_{k^*}>0$; then, informally, in order for the *k*-th clone ($k>k^*$) to become dominant, we must have $\log W_{k} - \log W_{k^*} \gtrapprox \alpha (T_k-T_{k^*})\simeq R(T_k)-R(T_k^*)$; in particular, if the immigration process is time-homogeneous, the increment between $\log W_{k^*}$ and $\log W_{k}$ must grow linearly with $k-k^*$, requiring the distribution of $\log W_k$ (given $W_k>0$) to be heavy tailed, which is not possible since $W_k$ has finite moments (Harris 1963). Convergence of Simpson’s index to a strictly positive r.v. reflects stabilization of the composition of GC in terms of clonal families and that the established order and composition are GC-specific. Also, $\lim _{t\rightarrow \infty } S_Y(t)<1$ shows that GC do not converge toward monoclonality. The higher $\alpha$, the higher $S_Y(t)$ (i.e., the more concentrated the distribution of clone size). Convergence of the Bray–Curtis index to the same limit as for $\textbf{Z}(\cdot )$ established in Proposition 2 arises from the fact that all clones obey the same offspring and lifetime distributions. Convergence of other beta diversity indices (to 0 or 1) captures absence of detectable temporal variation in clonal dominance.

### Differential fitness induces unstable clonal dominance

We now assume that $\{\alpha _k\}_{k=1}^\infty$ is a sequence of non-degenerate r.v., and show that any dominant clone is eventually outcompeted.

#### Proposition 4

Suppose that Assumption (A4) holds, $\lim \limits _{t\rightarrow \infty } R(t) = \infty$, $\textbf{P}\{\alpha _1>0\}>0$, $H_+(x)=\textbf{P}\{\alpha _1 \le x \vert \alpha _1>0\}$, $x\ge 0$, is absolutely continuous, and $\int _0^\infty g_k(x)^\delta dx <\infty$ for some $\delta >1$, $k=1,2\ldots$. Then, as $t\rightarrow \infty$, (i) $F_k(t){\mathop {\longrightarrow }\limits ^{a.s.}} 0$, and (ii) $\exists k' \in \{k+1,k+2\cdots \}$ a.s. such that $F_k(t)/F_{k'}(t){\mathop {\longrightarrow }\limits ^{a.s.}} 0$.

See Sect. 7.3 for a proof. Proposition 4 states that (*i*) the relative size of every clone within a GC becomes eventually negligible and (*ii*) every dominant clone is eventually outcompeted with probability one and regularly replaced as long as the GC reaction continues: an average of at least 3 weeks in lymph nodes and spleens, and much longer in Peyer’s patches. The replacement of dominant clones contributes to the diversification of the BCR repertoire by allowing new clones to expand over time. Informally, the replacement of a dominant clone occurs primarily when a new clone that joins the GC is not on a path to extinction and has a Malthusian parameter higher than that of the dominant clone. The phenomenon is also driven by a record process; however, unlike the one describes in Sect. 5.2, here the record process associates with the Malthusian sequence: $\alpha (t)=\max \{\alpha _k,k \in \{1\dots ,\Pi (t)\}: W_k>0\}$, $t\ge 0$. Let *L*(*t*) denote the number of such record values between times 0 and *t*. Since the number of clones joining the GC between the *L*(*t*)-th and $(L(t)+1)$-th records tends to increase exponentially with *L*(*t*) (Neuts 1967), the rate of clonal turnover should rapidly slow down with *L*(*t*), unless, for instance, the Malthusian parameters of founder B cells visiting the GC increase stochastically over time. This scenario could potentially occur if the GC was to recruit B cells released by other concurrent GC, exhibiting a high affinity for any of the antigens driving the reaction.

**Fig. 7:**
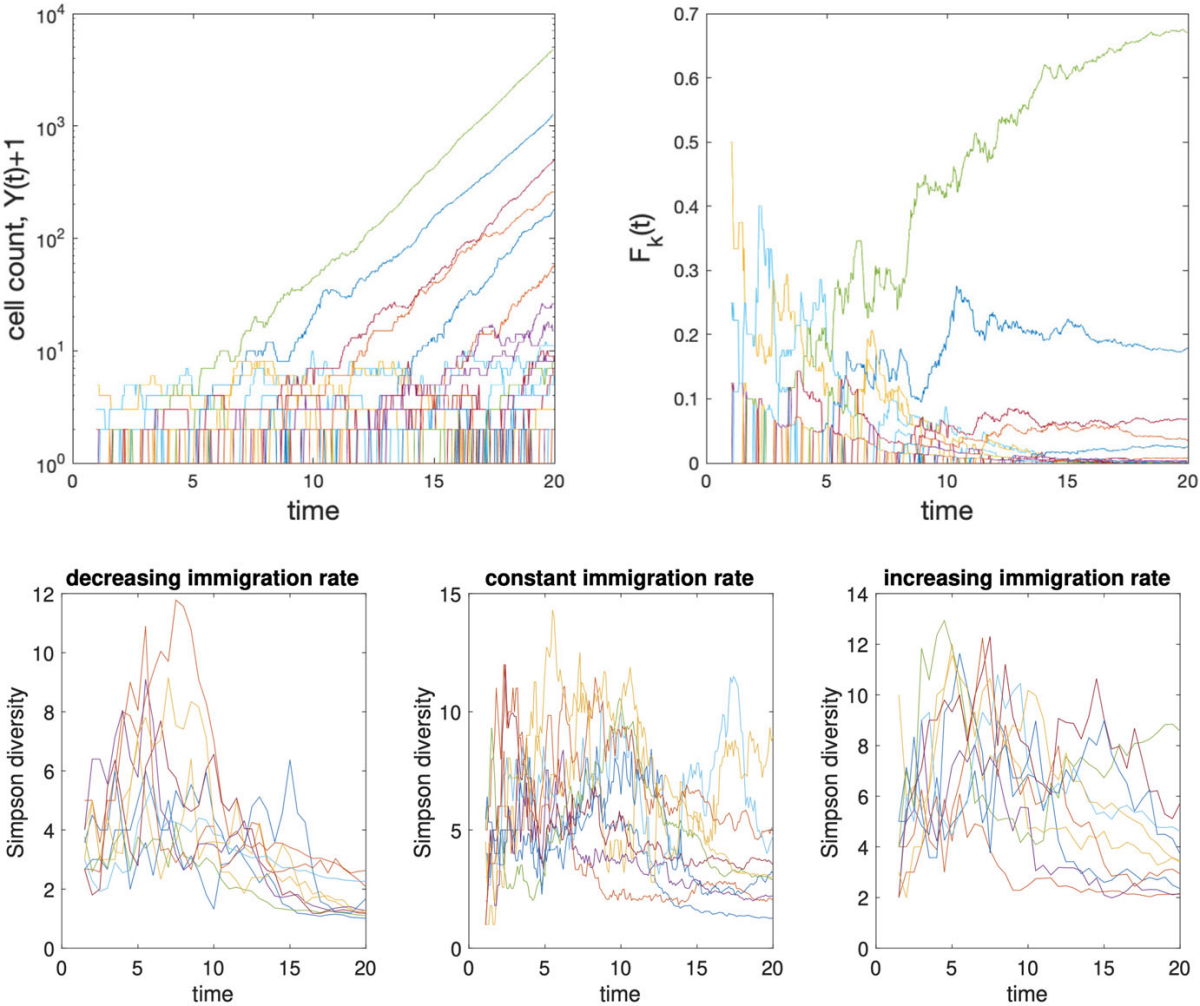
*Temporal evolution of clonal dominance and clonal diversity.* Top: Evolution of the absolute and relative size of individual clonal families (i.e., $Z_k^{(k)}(t)$ and $F_k(t)$) within a GC (each line represents one clonal family). Bottom: Evolution of clonal diversity within the GC, as measured by the Inverse Simpson index, in three scenarios corresponding to $r(t)=r_0 t^{-1/2}$ (left), $r(t)=r_0$ (center), and $r(t)=r_0t^{1/2}$ (right), with $r_0=10$ in all three cases. See Sect. 5.2 for detail

### Simulations: impact of the shape of immigration

We assumed that every $G_k(\cdot )$ is an exponential distribution with mean 1 so each unit of time represented the average duration of one mitotic cycle. For reference, the mean mitotic cycle of activated B cells has been estimated to last 8–10 h (Chan et al. 2021). We also assumed that $p_0^k$ was uniformly distributed in the interval [0.35, 1]. Thus, each process $Z_k^{(k)}(\cdot )$ was super-critical with probability 0.23 ($=0.15/0.65$). We were interested in studying the impact of the shape of the immigration rate on the dynamics of clonal families, considering three scenarios with different immigration rates: (1) $r(t)=r_0t^{-1/2}$, where immigration intensity decreases at a rate of $-1/2$ as discussed in Sect. 3.3; (2) $r(t)=r_0$, with a constant rate over time; and (3) $r(t)=r_0t^{1/2}$, where immigration intensity increases at a rate of $\gamma = 1/2$. The immigration *r*(*t*) has not yet been estimated based on experimental data. However, in the stationary case, it seems reasonable to assume that it is at least one order of magnitude faster than the mean mitotic cycle duration. Consequently, we set $r_0=10$ in all three cases. For computational reasons, we simulated the process over 20 units of time. Assuming that mitotic cycles and GC reactions last on average 10 h and 3 weeks, respectively, a duration of 20 units of time would cover almost half of the lifespan of a GC. We simulated the process 10 times in each scenario to identify patterns in the temporal trajectories of the Simpson index.

Results from the simulations are presented in Fig. 7. The top two panels show the evolution of the absolute and relative size of individual clonal families. Over the 20 time units, three clonal families reached clonal dominance (right panel). The bottom three panels indicate that clonal diversity, as measured by the Simpson diversity index, increases initially before leveling off between 5 and 10 units of time after initiation of the reaction. This pattern holds in the three immigration scenarios. In scenario (1) where the immigration rate decreases, the Simpson index tends to gradually decrease thereafter, suggesting that clonal diversity consistently reduces as the influx of founder B cells slows down. In the other two scenarios, diversity appears to also decrease but at a slower rate or less consistently among simulations. These results illustrate the potential benefit in sustaining the influx of B cells into GC in order to increase clonal diversity.

### Biological relevance

Recent studies estimated that around a few hundreds of founder B cells colonize GC, clearing the field from a commonly held belief that GC are seeded by 2–10 B cells (Faro and Or-Guil 2013; Tas et al. 2016; Kroese et al. 1987). Our model leads to a similar conclusion and supports the hypothesis that oligoclonality results from differential binding affinity between clones. We note that past studies were based on cross-sectional data. They could not track the number of clones accumulated in GC over their entire lifespan and may not have accounted for those that were outcompeted and eliminated before the GC were observed and for clones that had not yet joined the GC. Therefore, reported estimates may represent lower bounds for the number of founder B cells that join GC, and their actual number could be larger. Proposition 4 also suggests that clones with the highest binding affinity will tend to arrive late in GC, such that clonal dominance at a particular time point need not reflect optimal binding affinity.

## Convergent evolution within BCR repertoires

### Steering the repertoire via sequential immunization

Antibodies play a key role in protective immunity against various pathogens. Although only a handful of people are known to have been cured from HIV thus far, numerous studies have demonstrated that the bnAbs that emerge in a significant proportion of individuals with HIV infection can neutralize a wide array of circulating viral strains by targeting conserved HIV *epitopes*, the specific sites on the antigen that these bnAbs bind to, marking a promising avenue for potential therapeutic interventions. Particularly, a successful vaccine regimen might be designed by recapitulating the process giving rise to bnAbs via a sequence of strategically designed prime-boost immunogens that can trigger the activation of B cells with the molecular and genetic traits of targeted bnAbs and guide a subset of the B-cell repertoire toward neutralization against HIV by selecting appropriate somatic hypermutations. Figure 1 provides an illustration of the principle of this strategy assuming two boosts; a successful vaccine might require a larger number of boosts.

Two obstacles to the elicitation of bnAbs through vaccination are the exceptionally high mutation frequency characteristic of HIV bnAb which vaccines may have to induce and the difficulty of the germline precursors of HIV bnAbs to bind with the envelopes of circulating viral strains. In addition, some mutations necessary for neutralization may be rare, and the process of somatic hypermutation can either lead to mutations at positions where the bnAbs align with the germline or erase previously acquired mutations conducive to bnAb development. Neither of these outcomes may be desirable as they can impede or prevent maturation towards neutralization.

To quantify the process of steering BCR towards bnAbs, we introduce a model that describes the accumulation of somatic hypermutations in BCR relative to their germline and the targeted bnAb. To fix ideas, our model focuses on the region encoded by the V gene, also known as the V segment.

### Foreword to the modeling approach

In HIV vaccine research, evolution of the B-cell repertoire may be assessed by sequencing the heavy and light chains of thousands of antigen-specific B cells and comparing the mutations accumulated in their Ig gene loci with those found in relevant bnAbs. Multiple metrics may be designed to quantify this evolution in individual BCR sequences, depending in part on the class of B cells that the vaccine is expected to induce. Here, we opt for a simple, generic metric that enumerates mutations in five distinct regions of the heavy chain V segment. These regions are defined by whether mutations occur at positions where the aligned bnAb and germline gene sequences match or do not match. The proposed metric can be restricted to specific positions of the V segment without altering the model structure and interpretation.

Acknowledging the intricate nature of somatic hypermutation and antigen-mediated selection, we adopt a high-level modeling approach: instead of detailing BCR maturation on a per-position and per-nucleotide basis, we focus on counting the number of amino acid substitutions within the five identified regions. On one hand, aggregating multiple positions and nucleotide substitutions obscures the ability to specify how the accumulation of mutations alters antigen-mediated selection or the fitness of B cells. On the other hand, aggregation is expected to average out the impact of mutations on selection of B cells by the vaccine, at the level at which assumptions are postulated.

Our model offers a useful framework to understand some aspects of the evolution of the BCR repertoire. We use it in Sect. 6.7 to interpret BCR sequencing data. The analysis underscores the potential challenges in precisely replicating a particular antibody. Regardless, achieving neutralization through vaccination might not necessarily require reproducing a specific bnAb, as other unidentified antibodies could also induce broad neutralization.

### Partitioning the V segment into 5 classes of positions

**Fig. 8:**
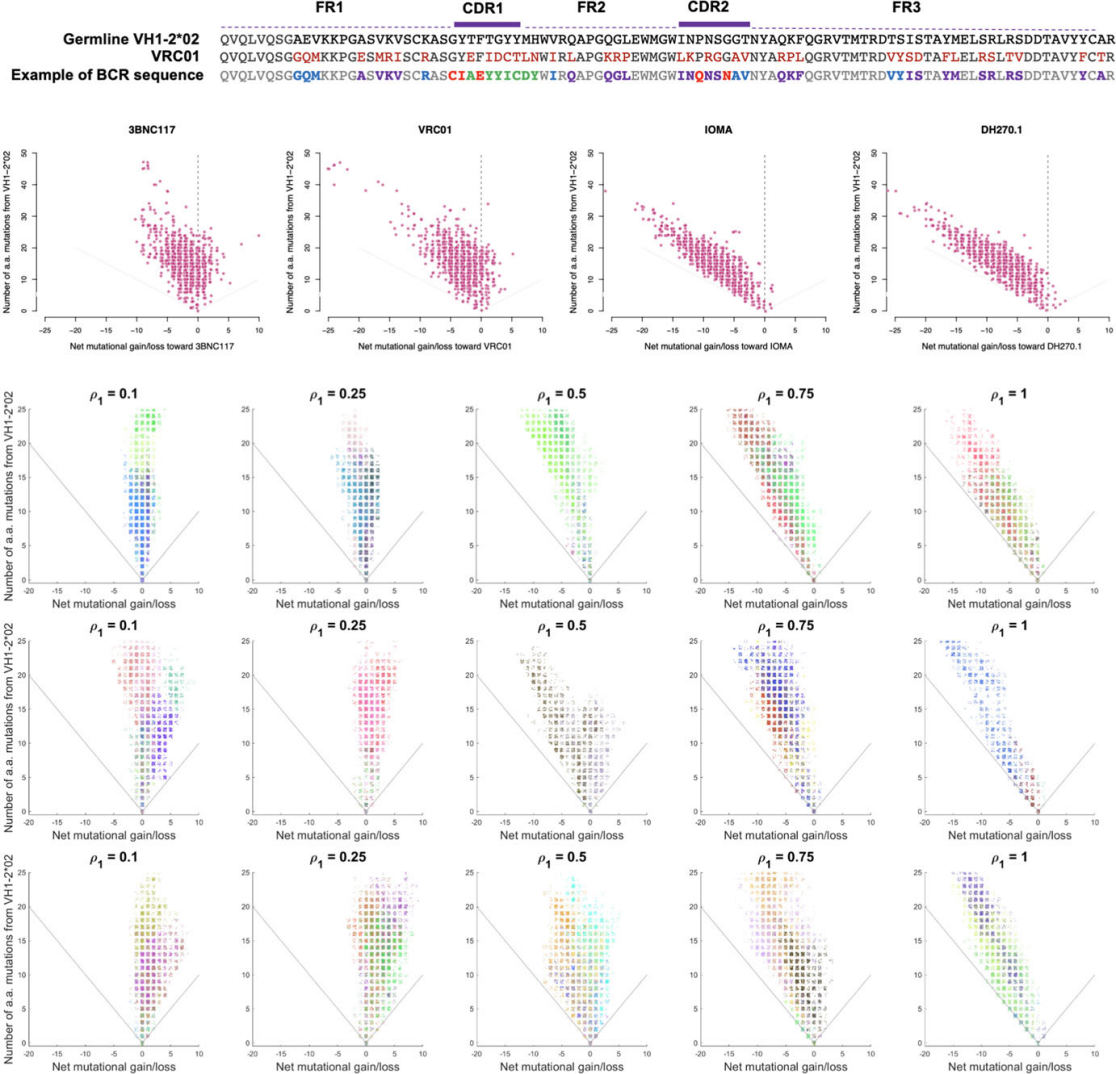
*Top row:* Amino acid sequences of the VH1-2*02 gene, V gene-encoded region of the heavy chain of VRC01 and of an arbitrary BCR. The 41 amino acids of VRC01 that differ from those of the germline VH1-2*02 gene form the subset $\mathcal {V}_2$ and are painted in red; the remaining 57 amino acids (black) define $\mathcal {V}_1$. In the BCR sequence example, grey amino acids define $\mathcal {V}_{11}(\textbf{S})$, those painted in *red* define $\mathcal {V}_{12}(\textbf{S})$, those in *purple* define $\mathcal {V}_{21}(\textbf{S})$, those in *blue* define $\mathcal {V}_{22}(\textbf{S})$, and those in *green* define $\mathcal {V}_{23}(\textbf{S})$. See Sect. 6.3 for a definition of these subsets. *Second row:* Number of mutated amino acids versus net mutational gain/loss in BCR sequences sampled from the B cell repertoire of several individuals, compared to four HIV-1 bnAbs (3BNC117, VRC01, IOMA, DH270.1) encoded by VH1-2*02. *Third to fifth row:* Number of mutated amino acids versus net mutational gain/loss in individual cells for several values of $\rho _1$ from simulations of a branching process. Each dot represents one cell or BCR, and each color identifies one clonal family. We set $\sigma _{12\vert 1} = \frac{1}{10}$ in row 3, $\sigma _{12\vert 1} = \frac{1}{2}$ in row 4, $\sigma _{12\vert 1} = \frac{4}{5}$ in row 5. See Sect. 6.7 for detail (color figure online)

Let $\textbf{B} = (B_1,\dots ,B_{L})$ and $\textbf{G} = (G_1,\dots ,G_L)$ denote the amino acid sequence of the V segment of a given targeted antibody and its germline V gene. To fix ideas, suppose that the antibody is VRC01; its heavy chain is encoded by the VH1-2*02 allele (Fig. 8). Let $\mathcal {V}$ denote the set of amino acid positions of the bnAb V segment. This set may be partitioned into two subsets: the positions at which the germline and bnAb amino acids are identical and those at which they differ. We respectively denote these two subsets by $\mathcal {V}_1$ and $\mathcal {V}_2$; they satisfy $\mathcal {V}_1 \cup \mathcal {V}_2 = \mathcal {V}$ and $\mathcal {V}_1 \cap \mathcal {V}_2 = \emptyset$. Let $L = \vert \mathcal {V}\vert$ denote the length of the V segment, and put $L_a = \vert \mathcal {V}_a\vert$, $a=1,2$. For VRC01, $L=98$, $L_1 = 57$, and $L_2=41$.

Now, let $\textbf{S}= (S_1,\dots ,S_{L'})$ denote the amino acid sequence of any BCR, to be compared against both its germline V gene and the bnAb. Let $\mathcal {V}_{11}(\textbf{S}) = \{ p \in \mathcal {V}_1: S_p = G_p\}$ denote the set of positions within $\mathcal {V}_1$ at which $\textbf{S}$ matches the germline and the bnAb. Define also $\mathcal {V}_{12}(\textbf{S}) = \{ p \in \mathcal {V}_1: S_p \ne G_p\} = \mathcal {V}_1 {\setminus } \mathcal {V}_{11}(\textbf{S})$, $\mathcal {V}_{21}(\textbf{S}) = \{ p \in \mathcal {V}_2: S_p = G_p\}$, $\mathcal {V}_{22}(\textbf{S}) = \{ p \in \mathcal {V}_2: S_p = B_p\}$, and $\mathcal {V}_{23}(\textbf{S})= \{ p \in \mathcal {V}_2: S_p \ne G_p, S_p \ne B_p\} = \mathcal {V}_2 {\setminus } ( \mathcal {V}_{21}(\textbf{S})\cup \mathcal {V}_{22}(\textbf{S}) )$. The mutations accumulated in $\textbf{S}$ that moved the BCR away from the bnAb are identified by $\mathcal {V}_{12}(\textbf{S})$, those shared with the bnAb belong to $\mathcal {V}_{22}(\textbf{S})$, whereas those that moved the BCR sequence $\textbf{S}$ away from the germline V gene without changing its Hamming distance to the bnAb are in $\mathcal {V}_{23}(\textbf{S})$. See Fig. 8 for an illustrative example. Mutations may change the size of the subsets $\mathcal {V}_{ab}(\textbf{S})$; however, by construction, the subsets $\mathcal {V}_{2b}(\textbf{S})$, $b=1,2,3$ are invariant to mutations in $\mathcal {V}_1$; likewise, mutations in $\mathcal {V}_2$ do not change the subsets $\mathcal {V}_{11}(\textbf{S})$ and $\mathcal {V}_{12}(\textbf{S})$.

Define ${L} (\textbf{S}) = (L_{11} (\textbf{S}),L_{12}(\textbf{S}),L_{21}(\textbf{S}),L_{22}(\textbf{S}),L_{23}(\textbf{S}))$ where $L_{ab} (\textbf{S}) = \vert \mathcal {V}_{ab}(\textbf{S}) \vert$ is the size (number of positions) in $\mathcal {V}_{ab}(\textbf{S})$, $(a,b) \in \{(1,1), (1,2),$ $(2,1),(2,2), (2,3)\}$. The total number of mutations in $\textbf{S}$ is $N(\textbf{S})= {L}_{12}(\textbf{S}) + {L}_{22}(\textbf{S}) + {L}_{23}(\textbf{S})$. A successful sequential immunization regimen might elicit BCR getting closer to targeted bnAbs as they accumulate mutations. A simple measure of the immune response toward this goal is the net mutational gain/loss relative to a bnAb, defined as $\Delta (\textbf{S}) = {L}_{22}(\textbf{S})- {L}_{12}(\textbf{S})$, with a positive value indicating overall progression of the sequence toward the target antibody. The dynamic model formulated in the next three sections describes the evolution of ${L} (\textbf{S})$ when $\textbf{S}$ is sampled from a tree generated by a multitype branching process.

### The mutational model, absent of cell kinetics

Let $\textbf{S}^{(0)}=(S^{(0)}_1,\dots , S^{(0)}_L)$ denote the amino acid sequence of the V segment of the BCR of any B cell at birth. We make the simplifying assumption that the two daughters of a dividing B cell inherit identical Ig gene sequences from their mother at division, represented by $\textbf{S}^{(1)}=(S^{(1)}_1,\dots , S^{(1)}_L)$. Unless a synonymous mutation occurs, the sequences $\textbf{S}^{(0)}$ and $\textbf{S}^{(1)}$ differ at the mutated position. Let $\tilde{\pi }$ denote the probability that mutations occur in the V segment. Allen et al. (1987) estimated that the BCR of one out of every other B cell inherits one mutation from their mother (Allen et al. 1987), such that we may set $\tilde{\pi }\in (0; 0.5)$ when focusing on the V segment. In what follows, we formulate a mutational model that describes the accumulation of somatic hypermutations in the subsets $\mathcal {V}_{ab}$, assuming at most one mutation happens per cell cycle.

For $a=1,2$, let $\pi _a^{\textbf{S}^{(0)}}$ denote the conditional probability that a mutation occurs in ${\textbf{S}^{(0)}}$ at one position in $\mathcal {V}_a$, given a mutation occurs. Mutations arise according to the following 10 scenarios (cases):

$$\begin{aligned} {L}(\textbf{S}^{(1)}) = {L}(\textbf{S}^{(0)}) + {\left\{ \begin{array}{ll} \textbf{e}_{1 \vert 1} := \begin{pmatrix} -1 &{} 1 &{} 0 &{} 0 &{}0 \end{pmatrix} &{} \hspace{-7.11317pt}\text{ w.p. } \pi _{1}^{\textbf{S}^{(0)}}\pi _{1 \vert 1}^{\textbf{S}^{(0)}} \hspace{59.75095pt} \text{ Case } \text{1.1 }\\ \textbf{e}_{2\vert 1} := \begin{pmatrix} 1 &{} -1 &{} 0 &{} 0 &{}0 \end{pmatrix} &{}\hspace{-7.11317pt}\text{ w.p. } \pi _{1}^{\textbf{S}^{(0)}}\pi _{2\vert 1}^{\textbf{S}^{(0)}} \hspace{59.75095pt} \text{ Case } \text{1.2 } \\ \textbf{e}_{3\vert 1} := \begin{pmatrix} 0 &{} 0 &{} 0 &{} 0 &{}0 \end{pmatrix} &{} \hspace{-7.11317pt}\text{ w.p. } \pi _{1}^{\textbf{S}^{(0)}}(1-\pi _{1\vert 1}^{\textbf{S}^{(0)}}-\pi _{2\vert 1}^{\textbf{S}^{(0)}}) \hspace{1.42271pt} \text{ Case } \text{1.3 }\\ \textbf{e}_{1\vert 2} := \begin{pmatrix} 0 &{} 0 &{} -1 &{} 1 &{} 0 \end{pmatrix} &{} \hspace{-7.11317pt}\text{ w.p. } \pi _{2}^{\textbf{S}^{(0)}}\pi _{1\vert 2}^{\textbf{S}^{(0)}} \hspace{59.75095pt} \text{ Case } \text{2.1 } \\ \textbf{e}_{2\vert 2} := \begin{pmatrix} 0 &{} 0 &{} -1 &{} 0 &{} 1 \end{pmatrix} &{} \hspace{-7.11317pt}\text{ w.p. } \pi _{2}^{\textbf{S}^{(0)}}\pi _{2\vert 2}^{\textbf{S}^{(0)}} \hspace{59.75095pt} \text{ Case } \text{2.2 } \\ \textbf{e}_{3\vert 2} := \begin{pmatrix} 0 &{} 0 &{} 1 &{} -1 &{} 0 \end{pmatrix} &{} \hspace{-7.11317pt}\text{ w.p. } \pi _{2}^{\textbf{S}^{(0)}}\pi _{3\vert 2}^{\textbf{S}^{(0)}} \hspace{59.75095pt} \text{ Case } \text{2.3 } \\ \textbf{e}_{4\vert 2} := \begin{pmatrix} 0 &{} 0 &{} 0 &{} -1 &{} 1 \end{pmatrix} &{} \hspace{-7.11317pt}\text{ w.p. } \pi _{2}^{\textbf{S}^{(0)}}\pi _{4\vert 2}^{\textbf{S}^{(0)}} \hspace{59.75095pt} \text{ Case } \text{2.4 } \\ \textbf{e}_{5\vert 2} := \begin{pmatrix} 0 &{} 0 &{} 1 &{} 0 &{}-1 \end{pmatrix} &{}\hspace{-7.11317pt}\text{ w.p. } \pi _{2}^{\textbf{S}^{(0)}}\pi _{5\vert 2}^{\textbf{S}^{(0)}} \hspace{59.75095pt} \text{ Case } \text{2.5 } \\ \textbf{e}_{6\vert 2} := \begin{pmatrix} 0 &{} 0 &{} 0 &{} 1 &{} -1 \end{pmatrix} &{} \hspace{-7.11317pt}\text{ w.p. } \pi _{2}^{\textbf{S}^{(0)}}\pi _{6\vert 2}^{\textbf{S}^{(0)}} \hspace{59.75095pt} \text{ Case } \text{2.6 } \\ \textbf{e}_{7\vert 2} : = \begin{pmatrix} 0 &{} 0 &{} 0 &{} 0 &{}0 \end{pmatrix}&\hspace{-7.11317pt}\text{ w.p. } \pi _{2}^{\textbf{S}^{(0)}}(1-\sum _{i=1}^6 \pi _{i\vert 2}^{\textbf{S}^{(0)}}) \hspace{8.5359pt} \text{ Case } \text{2.7 } \end{array}\right. } \end{aligned}$$

where $\pi _{i\vert a}^{\textbf{S}^{(0)}}$ denotes the conditional probability that a mutation occurs in $\textbf{S}^{(0)}$ according to Case a.i, given that a mutation occurs in $\mathcal {V}_a$. They reflect the impact of somatic hypermutation, accounting for both synonymous and non-synonymous mutations. Put $\pi _{3\vert 1}^{\textbf{S}^{(0)}} = 1-\sum _{i=1}^2 \pi _{i\vert 2}^{\textbf{S}^{(0)}}$ and $\pi _{7\vert 2}^{\textbf{S}^{(0)}} = 1-\sum _{i=1}^6 \pi _{i\vert 2}^{\textbf{S}^{(0)}}$.

In Case 1.1, a non-synonymous mutation in $\mathcal {V}_{11}(\textbf{S}^{(0)})$ moves the BCR further from the germline and bnAb. The mutation causes a one-position decrease in the size of $\mathcal {V}_{11}(\textbf{S}^{(0)})$ and a one-position increase in that of $\mathcal {V}_{12}(\textbf{S}^{(0)})$, as indicated by the increment $\textbf{e}_{1 \vert 1}=(-1,1,0,0,0)$. In Case 1.2, a non-synonymous mutation in $\mathcal {V}_{12}(\textbf{S}^{(0)})$ reverts a prior mutation back to the germline amino acid; hence, the change of $\textbf{e}_{2 \vert 1}=(1,-1,0,0,0)$. In Case 1.3, a mutation occurs in $\mathcal {V}_{1}$ without altering either $\mathcal {V}_{11}(\textbf{S}^{(0)})$ or $\mathcal {V}_{12}(\textbf{S}^{(0)})$. Hence, the absence of any difference between $\textbf{S}^{(0)}$ and $\textbf{S}^{(1)}$ indicated by $\textbf{e}_{3 \vert 1}=(0,0,0,0,0)$. Such mutations include synonymous mutation occurring in $\mathcal {V}_{11}(\textbf{S}^{(0)})$ and any mutations occurring in $\mathcal {V}_{12}(\textbf{S}^{(0)})$ that do not revert to the germline/bnAb amino acid.

In Case 2.1, a non-synonymous mutation in $\mathcal {V}_{21}(\textbf{S}^{(0)})$ matches the bnAb amino acid at the mutating position, bringing the BCR one amino acid closer to the bnAb. In Case 2.2, a non-synonymous mutation also in $\mathcal {V}_{21}(\textbf{S}^{(0)})$ does not match the bnAb amino acid at the mutating position and grows $\mathcal {V}_{23}(\textbf{S}^{(0)})$ by one position. In Case 2.3, a non-synonymous mutation in $\mathcal {V}_{22}(\textbf{S}^{(0)})$ reverts the BCR to the germline amino acid. In Case 2.4, a non-synonymous mutation occurs in $\mathcal {V}_{22}(\textbf{S}^{(0)})$ which does not match the germline amino acid. In Case 2.5, a non-synonymous mutation in $\mathcal {V}_{23}(\textbf{S}^{(0)})$ reverts the BCR back to the germline amino acid. In Case 2.6, a non-synonymous mutation also in $\mathcal {V}_{23}(\textbf{S}^{(0)})$ matches the bnAb amino acid. In Case 2.7, a mutation, possibly synonymous, in $\mathcal {V}_{2}$ matches neither the germline nor the bnAb amino acid, leaving the subsets $\mathcal {V}_{21}(\textbf{S}^{(0)})$, $\mathcal {V}_{22}(\textbf{S}^{(0)})$, and $\mathcal {V}_{23}(\textbf{S}^{(0)})$ unchanged. We note that mutations within $\mathcal {V}_{21}(\textbf{S}^{(0)})$ and $\mathcal {V}_{22}(\textbf{S}^{(0)})$ must necessarily be synonymous, whereas those within $\mathcal {V}_{23}(\textbf{S}^{(0)})$ can be either synonymous or non-synonymous.

### A parametrization of the transition probabilities

We present a parametrization of the probabilities $\pi _{i}^{\textbf{S}^{(0)}}$ and $\pi _{i\vert a}^{\textbf{S}^{(0)}}$ structured around the number of positions in $\mathcal {V}_{ab}(\textbf{S}^{(0)})$ to facilitate their interpretation and selecting their values in simulations. We note that, depending on $\textbf{S}^{(0)}$, not all 10 transitions presented in Sect. 6.4 are possible; for instance, Case 1.1 cannot happen when ${S}^{(0)}_1=0$. These cases are handled by setting $\pi _{i \vert a}^{\textbf{S}^{(0)}}=0$.

The probability of a mutation occurring in $\mathcal {V}_{1}$ compared to $\mathcal {V}_{2}$ may depend on the relative sizes of these two sets, and we write $\pi _1^{\textbf{S}^{(0)}} = \rho _1 \frac{ L_1 }{ L}=1-\pi _2^{\textbf{S}^{(0)}}$ for some constant $\rho _1\in (0,\frac{L}{L_1})$ which represents the selection bias in favor of ($\rho _1\in (1,L_1/L)$) or against $(\rho _1\in (0,1))$ bnAb-like mutations. When $\rho _1=1$, the odds of a mutation occurring in $\mathcal {V}_1$ instead of $\mathcal {V}_2$ is $\frac{L_1}{L_2}$ and depends solely on the size of $\mathcal {V}_1$ relative to that of $\mathcal {V}_2$. Since mutation rates vary across positions along the Ig gene sequences, positions in $\mathcal {V}_1$ may collectively have a higher (or lower) probability of mutation than those in $\mathcal {V}_2$, and we can adjust $\rho _1 \in (1, \frac{L}{L_1})$ to increase the likelihood of the mutation occurring in $\mathcal {V}_1$ relative to $\mathcal {V}_2$, and vice versa when $\rho _1\in (0,1)$.

To specify the probabilities $\pi _{i\vert a}^{\textbf{S}^{(0)}}$, it will be convenient to define the events $\mathcal {M}_a = \{\text{ a } \text{ mutation } \text{ occurs } \text{ in } \mathcal {V}_a\}$, $\mathcal {M}_{ab}^{\textbf{S}^{(0)}} = \{\text{ a } \text{ mutation } \text{ occurs } \text{ in } \mathcal {V}_{ab}(\textbf{S}^{(0)})\}$, and $\mathcal {S}_{ab}^{\textbf{S}^{(0)}} = \{\text{ the } \text{ mutated } \text{ sequence } {} \textbf{S}^{(1)}{} \text{ belongs } \text{ to } \mathcal {V}_{ab}(\textbf{S}^{(0)})\}$. Then, for mutations occurring in $\mathcal {V}_{1}$, we have that $\pi _{1\vert 1}^{\textbf{S}^{(0)}} = \mathbb {P} \big \{ \mathcal {M}_{11}^{\textbf{S}^{(0)}}$, $\mathcal {S}_{12}^{\textbf{S}^{(0)}}\vert \mathcal {M}_1 \big \}$, $\pi _{2\vert 1}^{\textbf{S}^{(0)}} = \mathbb {P} \big \{ \mathcal {M}_{12}^{\textbf{S}^{(0)}}$, $\mathcal {S}_{11}^{\textbf{S}^{(0)}}\vert \mathcal {M}_1 \big \}$, $\pi _{3\vert 1}^{\textbf{S}^{(0)}} = \pi _{3\vert 1,1}^{\textbf{S}^{(0)}} + \pi _{3\vert 1,2}^{\textbf{S}^{(0)}}$ where $\pi _{3\vert 1,1}^{\textbf{S}^{(0)}} = \mathbb {P} \big \{ \mathcal {M}_{11}^{\textbf{S}^{(0)}}, \mathcal {S}_{11}^{\textbf{S}^{(0)}}\vert \mathcal {M}_1 \big \}$ and $\pi _{3\vert 1,2}^{\textbf{S}^{(0)}} = \mathbb {P} \big \{ \mathcal {M}_{12}^{\textbf{S}^{(0)}},\mathcal {S}_{12}^{\textbf{S}^{(0)}}\vert \mathcal {M}_1 \big \}$. Similarly, for mutations occurring in $\mathcal {V}_{2}$, we have that $\pi _{1\vert 2}^{\textbf{S}^{(0)}} = \mathbb {P} \big \{ \mathcal {M}_{21}^{\textbf{S}^{(0)}}$, $\mathcal {S}_{22}^{\textbf{S}^{(0)}}\vert \mathcal {M}_2 \big \}$, $\pi _{2\vert 2}^{\textbf{S}^{(0)}} = \mathbb {P} \big \{ \mathcal {M}_{21}^{\textbf{S}^{(0)}}$, $\mathcal {S}_{23}^{\textbf{S}^{(0)}}\vert \mathcal {M}_2 \big \}$, $\pi _{3\vert 2}^{\textbf{S}^{(0)}} = \mathbb {P} \big \{ \mathcal {M}_{22}^{\textbf{S}^{(0)}}$, $\mathcal {S}_{21}^{\textbf{S}^{(0)}}\vert \mathcal {M}_2 \big \}$, $\pi _{4\vert 2}^{\textbf{S}^{(0)}} = \mathbb {P} \big \{ \mathcal {M}_{22}^{\textbf{S}^{(0)}}$, $\mathcal {S}_{23}^{\textbf{S}^{(0)}}\vert \mathcal {M}_2 \big \}$, $\pi _{5\vert 2}^{\textbf{S}^{(0)}} = \mathbb {P} \big \{ \mathcal {M}_{23}^{\textbf{S}^{(0)}}$, $\mathcal {S}_{21}^{\textbf{S}^{(0)}}\vert \mathcal {M}_2 \big \}$, $\pi _{6\vert 2}^{\textbf{S}^{(0)}} = \mathbb {P} \big \{ \mathcal {M}_{23}^{\textbf{S}^{(0)}}$, $\mathcal {S}_{22}^{\textbf{S}^{(0)}}\vert \mathcal {M}_2 \big \}$, and $\pi _{7\vert 1}^{\textbf{S}^{(0)}} = \pi _{7\vert 1,1}^{\textbf{S}^{(0)}} + \pi _{7\vert 1,2}^{\textbf{S}^{(0)}} + \pi _{7\vert 1,3}^{\textbf{S}^{(0)}}$ where $\pi _{7\vert 2,1}^{\textbf{S}^{(0)}} = \mathbb {P} \big \{ \mathcal {M}_{21}^{\textbf{S}^{(0)}}$, $\mathcal {S}_{21}^{\textbf{S}^{(0)}}\vert \mathcal {M}_2 \big \}$, $\pi _{7\vert 2,2}^{\textbf{S}^{(0)}} = \mathbb {P} \big \{ \mathcal {M}_{22}^{\textbf{S}^{(0)}}$, $\mathcal {S}_{22}^{\textbf{S}^{(0)}}\vert \mathcal {M}_2 \big \}$, and $\pi _{7\vert 2,3}^{\textbf{S}^{(0)}} = \mathbb {P} \big \{ \mathcal {M}_{23}^{\textbf{S}^{(0)}}$, $\mathcal {S}_{23}^{\textbf{S}^{(0)}}\vert \mathcal {M}_2 \big \}$.

Following the reasoning used to specify $\pi _1^{\textbf{S}^{(0)}}$, we assume that

$$\begin{aligned} \mathbb {P}\big \{ \mathcal {M}_{11}^{\textbf{S}^{(0)}} \vert \mathcal {M}_1 \big \} = \rho _{1\vert 1}\frac{L_{11}(\textbf{S}^{(0)})}{L_1} = 1-\mathbb {P}\big \{ \mathcal {M}_{12}^{\textbf{S}^{(0)}} \vert \mathcal {M}_1 \big \}, \end{aligned}$$

for some constant $\rho _{1\vert 1} \in (0, \frac{L_1}{L_{11}(\textbf{S}^{(0)})})$ modulating the odds of a mutation occurring in $\mathcal {V}_{11}(\textbf{S}^{(0)})$ compared to $\mathcal {V}_{12}(\textbf{S}^{(0)})$, after accounting for the number of positions. The per-position mutation rate is greater in $\mathcal {V}_{11}(\textbf{S}^{(0)})$ than $\mathcal {V}_{12}(\textbf{S}^{(0)})$ when $\rho _{1\vert 1} \in (1, \frac{L_1}{L_{11}(\textbf{S}^{(0)})})$, equal when $\rho _{1\vert 1}=1$, and lower when $\rho _{1\vert 1} \in (0, 1)$.

Additionally, we set $\mathbb {P}\big \{ \mathcal {S}_{11}^{\textbf{S}^{(0)}} \vert \mathcal {M}_{11}^{\textbf{S}^{(0)}} \big \} = \sigma _{1\vert 1} \mathbb {P}\big \{ \mathcal {S}_{12}^{\textbf{S}^{(0)}} \vert \mathcal {M}_{11}^{\textbf{S}^{(0)}} \big \}$ where $\sigma _{1\vert 1}$ represents the odds of a mutation occurring in $\mathcal {V}_{11}(\textbf{S}^{(0)})$ being synonymous. Most nucleotide substitutions of the third base (wobble position) of every codon induce synonymous mutations, whereas substitutions in the first and second bases induce non-synonymous mutations; hence, we set $\sigma _{1 \vert 1} = 1/3$ as an approximation in simulations. Similarly, we set $\mathbb {P}\big \{ \mathcal {S}_{12}^{\textbf{S}^{(0)}} \vert \mathcal {M}_{12}^{\textbf{S}^{(0)}} \big \} = \sigma _{2\vert 1} \mathbb {P}\big \{ \mathcal {S}_{11}^{\textbf{S}^{(0)}} \vert \mathcal {M}_{12}^{\textbf{S}^{(0)}} \big \}$ where $\sigma _{2\vert 1}$ represents the odds of a mutation occurring in $\mathcal {V}_{12}(\textbf{S}^{(0)})$ not reverting to the germline. We deduce that $\mathbb {P}\big \{ \mathcal {S}_{11}^{\textbf{S}^{(0)}} \vert \mathcal {M}_{11}^{\textbf{S}^{(0)}} \big \} = \frac{\sigma _{1\vert 1}}{1+ \sigma _{1\vert 1}}$ and $\mathbb {P}\big \{ \mathcal {S}_{12}^{\textbf{S}^{(0)}} \vert \mathcal {M}_{12}^{\textbf{S}^{(0)}} \big \} = \frac{\sigma _{2\vert 1}}{1+ \sigma _{2\vert 1}}$. Therefore, $\pi _{1\vert 1}^{\textbf{S}^{(0)}} = \frac{\rho _{1\vert 1}}{1+ \sigma _{1\vert 1}} \frac{L_{11}(\textbf{S}^{(0)})}{L_1}$, $\pi _{3\vert 1,1}^{\textbf{S}^{(0)}} = \frac{\sigma _{1\vert 1}\rho _{1 \vert 1}}{1+ \sigma _{1\vert 1}} \frac{L_{11}(\textbf{S}^{(0)})}{L_1}$, $\pi _{2\vert 1}^{\textbf{S}^{(0)}} = \frac{1}{1+ \sigma _{2\vert 1}} \left( 1- \rho _{1 \vert 1}\frac{L_{11}(\textbf{S}^{(0)})}{L_1} \right)$, and $\pi _{3\vert 1,2}^{\textbf{S}^{(0)}} = \frac{ \sigma _{2\vert 1} }{1+ \sigma _{2\vert 1}} \left( 1- \rho _{1 \vert 1}\frac{L_{11}(\textbf{S}^{(0)})}{L_1} \right)$.

In order to specify the probability of a mutation occurring in $\mathcal {V}_2$, we assume that mutations in $\mathcal {V}_{2}$ target $\mathcal {V}_{21}(\textbf{S}^{(0)})$ with probability $\pi _{1\vert 2}^{\textbf{S}^{(0)}} + \pi _{2\vert 2}^{\textbf{S}^{(0)}} + \pi _{7\vert 2,1}^{\textbf{S}^{(0)}} = \rho _{1\vert 2} \frac{L_{21}(\textbf{S}^{(0)})}{L_2}$, where the coefficient $\rho _{1\vert 2}$ modulates the per position mutation rate within $\mathcal {V}_{21}(\textbf{S}^{(0)})$ relative to $\mathcal {V}_{22}(\textbf{S}^{(0)}) \cup \mathcal {V}_{23}(\textbf{S}^{(0)})$. Likewise, mutations that occur in $\mathcal {V}_{2}$ target $\mathcal {V}_{22}(\textbf{S}^{(0)})$ with probability $\pi _{3\vert 2}^{\textbf{S}^{(0)}} + \pi _{4\vert 2}^{\textbf{S}^{(0)}} + \pi _{7\vert 2,2}^{\textbf{S}^{(0)}} = \rho _{2\vert 2} \frac{L_{22}(\textbf{S}^{(0)})}{L_2}$, whereas those that target $\mathcal {V}_{23}(\textbf{S}^{(0)})$ occur with probability $\pi _{5\vert 2}^{\textbf{S}^{(0)}} + \pi _{6\vert 2}^{\textbf{S}^{(0)}} + \pi _{7\vert 2,3}^{\textbf{S}^{(0)}} = \rho _{3\vert 2} \frac{L_{23}(\textbf{S}^{(0)})}{L_2}$. Since $\rho _{1\vert 2} \frac{L_{21}(\textbf{S}^{(0)})}{L_2}+\rho _{2\vert 2} \frac{L_{22}(\textbf{S}^{(0)})}{L_2}+\rho _{3\vert 2} \frac{L_{23}(\textbf{S}^{(0)})}{L_2}=1$, we deduce that

$$\begin{aligned} \rho _{3\vert 2} = \frac{L_2}{L_{23}(\textbf{S}^{(0)})} \left( 1 - \rho _{1\vert 2} \frac{L_{21}(\textbf{S}^{(0)})}{L_2} - \rho _{2\vert 2} \frac{L_{22}(\textbf{S}^{(0)})}{L_2} \right) \textbf{1}_{\{ L_{23}(\textbf{S}^{(0)}) >0\}}. \end{aligned}$$

We next assume that $\mathbb {P}\big \{ \mathcal {S}_{22}^{\textbf{S}^{(0)}} \vert \mathcal {M}_{21}^{\textbf{S}^{(0)}} \big \} = \sigma _{12\vert 2} \mathbb {P}\big \{ \mathcal {S}_{21}^{\textbf{S}^{(0)}} \vert \mathcal {M}_{21}^{\textbf{S}^{(0)}} \big \}$ and $\mathbb {P}\big \{ \mathcal {S}_{23}^{\textbf{S}^{(0)}} \vert \mathcal {M}_{21}^{\textbf{S}^{(0)}} \big \} = \sigma _{13\vert 2} \mathbb {P}\big \{ \mathcal {S}_{21}^{\textbf{S}^{(0)}} \vert \mathcal {M}_{21}^{\textbf{S}^{(0)}} \big \}$, where the parameters $\sigma _{12\vert 2}$ and $\sigma _{13\vert 2}$ modulate the odds of a mutation in $\mathcal {M}_{21}^{\textbf{S}^{(0)}}$ matching the target antibody sequence and matching neither the target antibody nor the germline sequences, respectively, relative to a mutation matching the germline sequence. Since $\mathbb {P}\big \{ \mathcal {S}_{21}^{\textbf{S}^{(0)}} \vert \mathcal {M}_{21}^{\textbf{S}^{(0)}} \big \} +\mathbb {P}\big \{ \mathcal {S}_{22}^{\textbf{S}^{(0)}} \vert \mathcal {M}_{21}^{\textbf{S}^{(0)}} \big \} +\mathbb {P}\big \{ \mathcal {S}_{23}^{\textbf{S}^{(0)}} \vert \mathcal {M}_{21}^{\textbf{S}^{(0)}} \big \} =1$, it follows that $\mathbb {P}\big \{ \mathcal {S}_{21}^{\textbf{S}^{(0)}} \vert \mathcal {M}_{21}^{\textbf{S}^{(0)}} \big \} = \frac{1}{1+\sigma _{12\vert 2}+\sigma _{13\vert 2}}$, $\mathbb {P}\big \{ \mathcal {S}_{22}^{\textbf{S}^{(0)}} \vert \mathcal {M}_{21}^{\textbf{S}^{(0)}} \big \} = \frac{\sigma _{12\vert 2}}{1+\sigma _{12\vert 2}+\sigma _{13\vert 2}}$, and $\mathbb {P}\big \{ \mathcal {S}_{23}^{\textbf{S}^{(0)}} \vert \mathcal {M}_{21}^{\textbf{S}^{(0)}} \big \} = \frac{\sigma _{13\vert 2}}{1+\sigma _{12\vert 2}+\sigma _{13\vert 2}}$.

Similarly, we assume that $\mathbb {P}\big \{ \mathcal {S}_{21}^{\textbf{S}^{(0)}} \vert \mathcal {M}_{22}^{\textbf{S}^{(0)}} \big \} = \sigma _{21\vert 2} \mathbb {P}\big \{ \mathcal {S}_{22}^{\textbf{S}^{(0)}} \vert \mathcal {M}_{22}^{\textbf{S}^{(0)}} \big \}$ and $\mathbb {P}\big \{ \mathcal {S}_{23}^{\textbf{S}^{(0)}} \vert \mathcal {M}_{22}^{\textbf{S}^{(0)}} \big \} = \sigma _{23\vert 2} \mathbb {P}\big \{ \mathcal {S}_{22}^{\textbf{S}^{(0)}} \vert \mathcal {M}_{22}^{\textbf{S}^{(0)}} \big \}$, where the parameters $\sigma _{21\vert 2}$ and $\sigma _{23\vert 2}$ modulate the odds of a mutation in $\mathcal {M}_{22}^{\textbf{S}^{(0)}}$ matching the germline sequence and matching neither the target antibody nor the germline sequences, respectively, relative to a mutation matching the bnAb sequence. Since $\mathbb {P}\big \{ \mathcal {S}_{21}^{\textbf{S}^{(0)}} \vert \mathcal {M}_{22}^{\textbf{S}^{(0)}} \big \} +\mathbb {P}\big \{ \mathcal {S}_{22}^{\textbf{S}^{(0)}} \vert \mathcal {M}_{22}^{\textbf{S}^{(0)}} \big \} +\mathbb {P}\big \{ \mathcal {S}_{23}^{\textbf{S}^{(0)}} \vert \mathcal {M}_{22}^{\textbf{S}^{(0)}} \big \} =1$, we deduce that $\mathbb {P}\big \{ \mathcal {S}_{21}^{\textbf{S}^{(0)}} \vert \mathcal {M}_{22}^{\textbf{S}^{(0)}} \big \} = \frac{\sigma _{21\vert 2}}{1+\sigma _{21\vert 2}+\sigma _{23\vert 2}}$, $\mathbb {P}\big \{ \mathcal {S}_{22}^{\textbf{S}^{(0)}} \vert \mathcal {M}_{22}^{\textbf{S}^{(0)}} \big \} = \frac{1}{1+\sigma _{21\vert 2}+\sigma _{23\vert 2}}$, and $\mathbb {P}\big \{ \mathcal {S}_{23}^{\textbf{S}^{(0)}} \vert \mathcal {M}_{22}^{\textbf{S}^{(0)}} \big \} = \frac{\sigma _{23\vert 2}}{1+\sigma _{21\vert 2}+\sigma _{23\vert 2}}$.

Finally, we assume that $\mathbb {P}\big \{ \mathcal {S}_{21}^{\textbf{S}^{(0)}} \vert \mathcal {M}_{23}^{\textbf{S}^{(0)}} \big \} = \sigma _{31\vert 2} \mathbb {P}\big \{ \mathcal {S}_{23}^{\textbf{S}^{(0)}} \vert \mathcal {M}_{23}^{\textbf{S}^{(0)}} \big \}$ and $\mathbb {P}\big \{ \mathcal {S}_{22}^{\textbf{S}^{(0)}} \vert \mathcal {M}_{23}^{\textbf{S}^{(0)}} \big \} = \sigma _{32\vert 2} \mathbb {P}\big \{ \mathcal {S}_{22}^{\textbf{S}^{(0)}} \vert \mathcal {M}_{23}^{\textbf{S}^{(0)}} \big \}$, where the parameters $\sigma _{31\vert 2}$ and $\sigma _{32\vert 2}$ modulate the odds of a mutation in $\mathcal {M}_{23}^{\textbf{S}^{(0)}}$ matching the germline sequence and matching neither the target antibody sequence, respectively, relative to a mutation matching the bnAb sequence. Since $\mathbb {P}\big \{ \mathcal {S}_{21}^{\textbf{S}^{(0)}} \vert \mathcal {M}_{23}^{\textbf{S}^{(0)}} \big \} +\mathbb {P}\big \{ \mathcal {S}_{22}^{\textbf{S}^{(0)}} \vert \mathcal {M}_{23}^{\textbf{S}^{(0)}} \big \} +\mathbb {P}\big \{ \mathcal {S}_{23}^{\textbf{S}^{(0)}} \vert \mathcal {M}_{23}^{\textbf{S}^{(0)}} \big \} =1$, we deduce that $\mathbb {P}\big \{ \mathcal {S}_{21}^{\textbf{S}^{(0)}} \vert \mathcal {M}_{23}^{\textbf{S}^{(0)}} \big \} = \frac{\sigma _{31\vert 2}}{1+\sigma _{31\vert 2}+\sigma _{32\vert 2}}$, $\mathbb {P}\big \{ \mathcal {S}_{22}^{\textbf{S}^{(0)}} \vert \mathcal {M}_{23}^{\textbf{S}^{(0)}} \big \} = \frac{\sigma _{32\vert 2}}{1+\sigma _{31\vert 2}+\sigma _{32\vert 2}}$, and $\mathbb {P}\big \{ \mathcal {S}_{23}^{\textbf{S}^{(0)}} \vert \mathcal {M}_{23}^{\textbf{S}^{(0)}} \big \} = \frac{1}{1+\sigma _{31\vert 2}+\sigma _{32\vert 2}}$.

Since $\pi _{1 \vert 2} = \mathbb {P}\{\mathcal {S}_{22}^{\textbf{S}^{(0)}}\vert \mathcal {M}_{21}^{\textbf{S}^{(0)}}\big \} \mathbb {P} \big \{ \mathcal {M}_{21}^{\textbf{S}^{(0)}} \vert \mathcal {M}_2 \}$, the above assumptions lead to $\pi _{1\vert 2} =\frac{ \rho _{1\vert 2} \sigma _{12\vert 2}}{1+\sigma _{12\vert 2}+\sigma _{13\vert 2}} \frac{L_{21}(\textbf{S}^{(0)})}{L_2}$. Likewise, $\pi _{2\vert 2}=\frac{ \rho _{1\vert 2} \sigma _{13\vert 2}}{1+\sigma _{12\vert 2}+\sigma _{13\vert 2}} \frac{L_{21}(\textbf{S}^{(0)})}{L_2}$, $\pi _{3\vert 2} =\frac{ \rho _{2\vert 2} \sigma _{21\vert 2}}{1+\sigma _{21\vert 2}+\sigma _{23\vert 2}} \frac{L_{22}(\textbf{S}^{(0)})}{L_2}$, $\pi _{4\vert 2}=\frac{ \rho _{2\vert 2} \sigma _{23\vert 2}}{1+\sigma _{21\vert 2}+\sigma _{23\vert 2}} \frac{L_{22}(\textbf{S}^{(0)})}{L_2}$, $\pi _{5\vert 2} =\frac{ \rho _{3\vert 2} \sigma _{31\vert 2}}{1+\sigma _{31\vert 2}+\sigma _{32\vert 2}} \frac{L_{23}(\textbf{S}^{(0)})}{L_2}$, $\pi _{6\vert 2} =\frac{ \rho _{3\vert 2} \sigma _{32\vert 2}}{1+\sigma _{31\vert 2}+\sigma _{32\vert 2}} \frac{L_{23}(\textbf{S}^{(0)})}{L_2}$, and $\pi _{7\vert 2} =1 - \sum _{i=1}^6 \pi _{i\vert 2}$.

### A branching process of convergent evolution

In a clone started from a naive B cell, the BCR sequence of the founder B cell is such that ${L}(\textbf{S})=(L_1,0,L_2,0,0)$ because it is unmutated. As cells divide, mutations accumulate in the clonal family, and BCR sequences $\textbf{S}$ accumulate mutations, and the collection of vectors ${L}(\textbf{S})$ represents the position of the clonal family with respect to the germline and bnAb sequences. Here we investigate whether the BCR repertoire converges to a bnAb, and whether convergence to a bnAb requires a highly favorable set of circumstances.

To describe the intra-clonal evolution of the distances ${L}(\textbf{S})$, we define a multitype age-dependent branching process in which every cell is assigned a type represented by a vector $\varvec{\ell }$ that takes values in the set

$$\begin{aligned} \mathcal {K} = \{ \varvec{\ell } = (\ell _1,\dots ,\ell _5) \in \mathbb {N}^5: \ell _1+\ell _2 = L_1; \ell _3+\ell _4+\ell _5 = L_2\}, \end{aligned}$$

where $\mathbb {N}=\{0,1\ldots \}$ represents the set of non-negative integers. The entries of $\varvec{\ell }$ are interpreted just as those of ${L}(\textbf{S})$, as described in Sect. 6.4.

For every $\big (\varvec{\ell }^{(0)},\varvec{\ell }^{(1)},\varvec{\ell }^{(2)} \big ) \in \mathcal {K} \times \mathcal {K} \times \mathcal {K}$, the conditional probability that any cell of type $\varvec{\ell }^{(0)}$ divides into one cell of type $\varvec{\ell }^{(1)}$ and one cell of type $\varvec{\ell }^{(2)}$, given it divides, is assumed to be

22 $$\begin{aligned} q_{\varvec{\ell }^{(1)}\varvec{\ell }^{(2)}}^{{\varvec{\ell }}^{(0)}} = {\left\{ \begin{array}{ll} 1-\tilde{\pi }&{} \text{ if } \text{ no } \text{ mutation } \text{ occurs, } \text{ and } \varvec{\ell }^{(1)}=\varvec{\ell }^{(2)} =\varvec{\ell }^{(0)} \\ \tilde{\pi }\pi _a^{\varvec{\ell }^{(0)}} \pi _{i \vert a }^{\varvec{\ell }^{(0)}} &{} \text{ if } \varvec{\ell }^{(1)}=\varvec{\ell }^{(2)} =\varvec{\ell }^{(0)} + \textbf{e}_{i \vert a} \\ 0 &{} \text{ otherwise. } \end{array}\right. } \end{aligned}$$

The first equation specifies the probability of no mutation occurring. The second one specifies the probability of a mutation occurring in $\mathcal {V}_a$ according to Case a.i, as described in Sect. 6.4, with both daughters inheriting the same set of mutations from their mother. The third equation reflects the assumption that both daughter cells cannot inherit different mutations from their mother.

We assume that the probability of death and the lifespan distribution of every cell are independent of its type: $p_0^{\varvec{\ell }} = p_0$ and $G_{\varvec{\ell }}(\cdot ) \equiv G(\cdot )$, $\varvec{\ell } \in \mathcal {K}$. This assumption is motivated by the aggregation of positions and amino acid substitutions in defining the five regions, as discussed in Sect. 6.2. Together with the conditional probabilities $q_{\varvec{\ell }^{(1)}\varvec{\ell }^{(1)}}^{ \varvec{\ell }^{(0)}}$, they model the combined outcome of mutation and antigen-mediated selection. Because it does not distinguish these two processes, the proposed model is partly phenomenological. The entries of $M = (m_{\varvec{\ell }^{(0)}\varvec{\ell }^{(1)}})_{\varvec{\ell }^{(0)},\varvec{\ell }^{(1)} \in \mathcal {K}}$ are given by $m_{\varvec{\ell }^{(0)} \varvec{\ell }^{(1)}} = 2(1-p_{0})q_{\varvec{\ell }^{(1)}\varvec{\ell }^{(1)}}^{ \varvec{\ell }^{(0)}}$, such that $M = 2(1-p_0)Q$ where $Q = (q_{\varvec{\ell }^{(1)}\varvec{\ell }^{(1)}}^{ \varvec{\ell }^{(0)}})_{\varvec{\ell }^{(0)},\varvec{\ell }^{(1)} \in \mathcal {K}}$.

#### Proposition 5

Suppose that *Q* is irreducible, $p_0<1/2$, and (A4) holds. Let $G^*(\cdot )$ and $G^{*,-1}(\cdot )$ denote the Laplace transform of $G(\cdot )$ and the inverse of $G^*(\cdot )$. Then, as $t\rightarrow \infty$, $e^{-\alpha t} \textbf{Z}(t) {\mathop {\longrightarrow }\limits ^{a.s.}} W \textbf{v}$ where $\alpha = G^{*,-1} \big (1/(2(1-p_0))\big )$ and $\textbf{v}=(v_{\varvec{\ell }})_{\varvec{\ell } \in \mathcal {K}}$ is the left eigenvector of *Q* associated with its eigenvalue 1 and such that $\sum _{\varvec{\ell } \in \mathcal {K}} v_{\varvec{\ell }}=K$.

See Sect. 7.4 for a proof. The irreducibility assumption on *Q* holds, for example, if mutations in $\mathcal {V}_1$ and $\mathcal {V}_2$ are reversible. It follows from Proposition 5 that, conditional on survival (i.e., $W>0$), the frequency of mutations within a clone converges a.s. over time; specifically,

23 $$\begin{aligned} \frac{\textbf{Z}(t)}{\sum _{\varvec{\ell } \in \mathcal {K}} Z_{\varvec{\ell }}(t)} = \frac{e^{-\alpha t} \textbf{Z}(t)}{\sum _{\varvec{\ell } \in \mathcal {K}} e^{-\alpha t} Z_{\varvec{\ell }}(t)} {\mathop {\longrightarrow }\limits ^{a.s.}} \frac{\textbf{v}}{K} \quad \text{ as } t\rightarrow \infty , \end{aligned}$$

such that the entries of the vector $\textbf{v}/K$ provide the asymptotic mean frequency of the number of positions of $\mathcal {V}$ falling into the subsets $\mathcal {V}_{ab}$, $(a,b) \in \{(1,1), (1,2),$ $(2,1),(2,2), (2,3)\}$, in expanded clones.

### Drift of the BCR repertoire relative to HIV bnAbs

We randomly selected 1000 BCR sequences sampled from human subjects, none of which were living with HIV (Guo et al. 2019). All BCR were inferred to be encoded by the VH1-2*02 allele. We calculated the number of mutations relative to VH1-2*02 and the net mutational gain/loss against four HIV bnAbs: VRC01, 3BNC117, IOMA, DH270.1, all originating from VH1-2*02 (Fig. 8, middle row). As expected, BCR induced by antigens unrelated to HIV preferentially accumulate mutations that are distinct from those observed in the HIV bnAbs, as evidenced by the amount of mutational losses increasing with the number of mutations. Thus, sequential HIV vaccines may have to select for rare mutations to steer antibodies toward bnAbs. The data also suggest that inducing bnAb-specific mutations is more challenging for some bnAbs than other, as indicated by the faster drift of some BCRs away from IOMA and DH270.1 relative to 3BNC117 and VRC01.

**Fig. 9:**
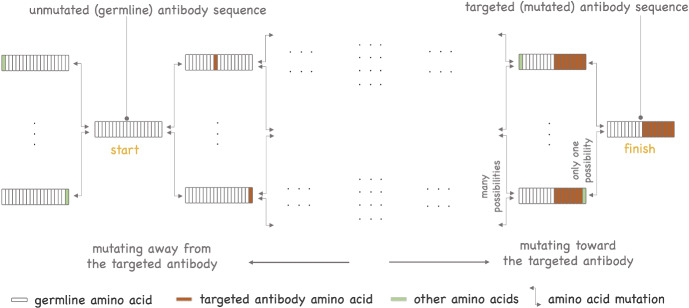
*Mutational paths toward a targeted antibody sequence.* Each horizontal block of adjacent vertical bars represents one antibody sequence, partitioned into nucleotide positions. Arrows represent one-nucleotide mutations between antibody sequences. This diagram gives a simplified overview of the sets of mutational paths induced by somatic hypermutations started from an unmutated (germline) antibody sequence toward a targeted antibody sequence. Progression toward the targeted antibody may be hampered by the fact that as sequences accumulate targeted mutations, the number of candidate unfavorable mutations increases relative to those that are favorable. When the antibody is one mutation away from the target, the remaining mutation is up against all other potential mutations. This challenge may be collectively addressed by the multiple B cells from a clonal family that may express identical or similar antibody sequences, each offering a chance of successfully mutating toward the targeted sequence

To interpret this observation, we used our model to first examine the evolution of the BCR repertoire relative to VRC01. We set the probability of death ($p_0$) to $\frac{4}{10}$ to ensure the survival of clonal families while preventing excessive expansion for computational feasibility. The V segment of heavy chains accounts for slightly less than half of the variable domain, but is somewhat more prone to somatic hypermutations than other parts of the BCR. Therefore, taking Allen et al. (1987)’s estimate of the mutation rate into consideration, we set the probability of mutation in the V segment ($\tilde{\pi }$) to $\frac{1}{4}$. The mutation rate may differ between $\mathcal {V}_1$ and $\mathcal {V}_2$, partly because some bnAb-specific mutations may be much less frequent than non-bnAb specific mutations. These probabilities also depend on the antigens to which B cells are exposed. Thus, we considered several values of $\rho _{1} = \frac{1}{10}, \frac{1}{4}, \frac{1}{2},\frac{3}{4},\frac{9}{10}$ to perform a sensitivity analysis. The BCR sequencing data used in our analysis resulted from exposures to highly diverse antigens. Therefore, we do not expect the per-position mutation rates to differ between the sets $\mathcal {V}_{ab}(\textbf{S}^{(0)})$, and put $\rho _{1\vert 1} =\rho _{1\vert 2}=\rho _{2\vert 2}=1$. As argued in Sect. 6.5, we set $\sigma _{1\vert 1}=\frac{1}{3}$ to specify the likelihood of a mutation in $\mathcal {V}_{11}(\textbf{S}^{(0)})$ being synonymous. To specify the probability of a mutation in $\mathcal {V}_{12}(\textbf{S}^{(0)})$ not reverting to a germline amino acid, we set $\sigma _{2 \vert 1}= \frac{1}{10}$. This choice is based on several considerations. Firstly, in the best-case scenario where reverting to a germline amino acid requires a single mutation, most substitutions at the wobble base, which may account for approximately one-third of all mutations, would not qualify. Among the remaining two-thirds of potential mutations at the first and second bases, only those that induce the specific germline amino acid would produce a reverse mutation, acknowledging however that the (effective) average number of candidate amino acid mutations is likely lower than 19 (ignoring mutations into stop codons). Furthermore, as mutations accumulate, some positions may require two or even more simultaneous mutations to revert to the germline amino acid. With an effective average number of five amino acid mutations, we might expect $\sigma _{2 \vert 1}\simeq \frac{2}{15}$ in the best-case scenario; accounting for cases requiring more than one mutations provides support for setting $\sigma _{2 \vert 1}= \frac{1}{10}$ or lower. We note that the choice of $\sigma _{2 \vert 1}$ has limited impact if $L_{12}(\textbf{S}^{(0)})/L_1$ is small. Following a similar line of arguments, we set $\sigma _{12 \vert 1}= \frac{1}{10}$ to specify the probability of a mutation in $\mathcal {V}_{12}(\textbf{S}^{(0)})$ matching the bnAb amino acid, acknowledging that a lower value may be more appropriate to study drift of the B cell repertoire following exposure to HIV-related antigens which may induce bnAb-like mutations with higher probability. Thus, we explored values of $\sigma _{12 \vert 1}= \frac{1}{5}$ and $\sigma _{12 \vert 1}= \frac{1}{2}$ in sensitivity analyses. Based on previous arguments regarding synonymous mutations, we set $\mathbb {P}\big \{ \mathcal {S}_{21}^{\textbf{S}^{(0)}} \vert \mathcal {M}_{21}^{\textbf{S}^{(0)}} \big \} =\frac{1}{3}$. Hence, $\mathbb {P}\big \{ \mathcal {S}_{22}^{\textbf{S}^{(0)}} \vert \mathcal {M}_{21}^{\textbf{S}^{(0)}} \big \} + \mathbb {P}\big \{ \mathcal {S}_{23}^{\textbf{S}^{(0)}} \vert \mathcal {M}_{21}^{\textbf{S}^{(0)}} \big \} =\frac{2}{3}$ which gives $\sigma _{13 \vert 2} = 2 - \sigma _{12 \vert 2}$ after algebraic calculations, and we set $\sigma _{13 \vert 2}=\frac{19}{10}$. Justified by a similar line of arguments, we set $\sigma _{21 \vert 2}=\frac{1}{10}$ and $\sigma _{23 \vert 2}=\frac{19}{10}$. Finally, we assume that $\sigma _{31 \vert 2} = \sigma _{32 \vert 2} = \frac{1}{10}$ using the line of arguments used to justify setting $\sigma _{12 \vert 2} = \frac{1}{10}$.

Results from simulations are displayed in Fig. 8, sorted by values of $\rho _1$ (columns) and $\sigma _{12 \vert 1}$ (rows). When $\sigma _{12 \vert 1}=\frac{1}{10}$, overall, patterns observed in simulations are similar to those from BCR sequencing data, indicating a tendency of the BCR repertoire to drift away from the bnAb as mutations accumulate in the V segment, unless $\rho _1$ is close to 0; for example, $\rho _1 \le \frac{1}{10}$, reflecting that the per-position mutation rate is at least 10 times higher in $\mathcal {V}_2$ than in $\mathcal {V}_1$. In the absence of selection bias ($\rho _1=1$), mutations would appear in $\mathcal {V}_2$ with a probability of $41.8\%$ $(=\frac{41}{98} \times 100$%). Thus, even with almost $50\%$ chance of inducing a mutation in $\mathcal {V}_2$, the BCR repertoire is expected to rapidly drift away from VRC01 (see panel with $\rho =1.0$) because the bnAb amino acid is only one of the candidate mutations. Our simulations suggest a value of $\rho _1\simeq 0.5$ for VRC01, indicating that affinity maturation during exposures to HIV-unrelated antigens has a preferential bias for inducing mutations at positions at which VRC01 and VH1-2*02 do not match, and that the probability of this happening is about twice as large compared to if there was no selection bias (i.e., case where $\rho _1=1$). The results obtained by increasing $\sigma _{12 \vert 1}$ to $\frac{1}{2}$ or $\frac{4}{5}$ suggest that this parameter has some but relatively limited influence on the results, with its impact decreasing with $\rho$.

The value of $\rho _1$ appeared to differ between the four bnAbs. After accounting for the number of mutations in their respective V segment, simulations suggested the following values of $\rho _1$: $\rho _1\simeq 0.5$ for 3BNC117 and $\rho _1 \ge 0.9$ for both IOMA and DH270.1. Thus, induction of mutations in $\mathcal {V}_2$ were less likely for IOMA and DH270.1 than for VRC01 and 3BNC117. One potential explanation is the fact that VRC01 and 3BNC117 are more mutated than IOMA and DH270.1, making the induction of VRC01- and 3BNC117-like mutations easier. Typically, as BCRs approach a targeted antibody, the challenge of accumulating additional advantageous mutations tends to increase because the probability of encountering unfavorable mutations eventually surpasses that of favorable ones, as illustrated in Fig. 9. This could result in a slowdown in progress as B cells approach the target. The point of equilibrium towards which members of a clonal family tend to gravitate is formally described in Proposition 5 and equation (23).

## Appendices: Proofs

### Proof of Proposition 2

We begin the proof with the Bray–Curtis index. Since Assumption (A4) holds and $W>0$, we have for $\delta \ge 0$ and *t* large enough that

$$\begin{aligned} BC_Z(t,t+\delta )= & {} \sum _{k=1}^K \frac{ |(v_k W e^{\alpha t} - v_k W e^{\alpha (t+\delta )})(1+o(1)) |}{ \sum _{k'=1}^K (v_{k'} W e^{\alpha t}+ v_{k'} W e^{\alpha (t+\delta )}) (1+o(1))} \\= & {} \sum _{k=1}^K \frac{ v_k |e^{\alpha t} - e^{\alpha (t+\delta )} |(1+|o (1) |) }{ \sum _{k'=1}^K v_{k'} e^{\alpha t} (1+e^{\alpha \delta }) (1+|o(1) |) } \\= & {} \sum _{k=1}^K \frac{ v_k |1 - e^{-\alpha \delta } |(1+|o(1) |) }{ \sum _{k'=1}^K v_{k'} (1+e^{\alpha \delta }) (1+|o(1) |) }. \end{aligned}$$

Therefore, a.s., $\lim \limits _{t\rightarrow \infty }BC_{Z}(t;t+\delta )=(1-e^{-\alpha \delta })/(1+e^{\alpha \delta })$. This establishes the first statement. Derivation of the other results of Proposition 2 uses similar arguments and are not provided.

### Proof of Proposition 3

We have for every $k=1,2\ldots$ that $\lim \nolimits _{t\rightarrow \infty } e^{-\alpha t} Z^{(k)}_k(t-T_k) = W_{k} c e^{-\alpha T_{k}}$ (a.s.) for some constant $c>0$. Let $W_k(u)=e^{-\alpha u} Z^{(k)}_k(u) / c$.

We first show that the series $\sum _{k=1}^{\infty } W_{k} c e^{-\alpha T_{k}}$ converges a.s. by verifying the conditions of Kolmogorov’s Three Series Theorem. Let $A>0$, arbitrary. To prove the first condition, notice that

$$\begin{aligned} \sum _{k=1}^{\infty } \mathbb {P} \{ W_{k} e^{-\alpha T_{k}} \ge A \}\le & {} \sum _{k=1}^{\infty } \frac{\mathbb {E} ( W_{k} e^{-\alpha T_{k}} ) }{A} = \frac{1}{A} \sum _{k=1}^{\infty } \mathbb {E} ( e^{-\alpha T_{k}} )\\= & {} \frac{1}{A} \sum _{k=1}^{\infty } \left( 1 + \frac{\alpha }{r_0} \right) ^{-k} \\= & {} \frac{1}{A} \left( \frac{1}{1 - \left( 1 + \frac{\alpha }{r_0} \right) ^{-1}} - 1 \right) = \frac{1}{A} \frac{r_0}{\alpha } \ < \ \infty , \end{aligned}$$

where we have used the assumption that $\alpha >0$. To prove the second condition, we have that

24 $$\begin{aligned} \sum _{k=1}^{\infty } \mathbb {E} ( W_{k} e^{-\alpha T_{k}} \textbf{1}_{\{ W_{k} e^{-\alpha T_{k}} \le A \}} ) \le \sum _{k=1}^{\infty } \mathbb {E} ( e^{-\alpha T_{k}} ) = \frac{r_0}{\alpha } < \infty . \end{aligned}$$

Lastly, the following inequality proves the third condition:

$$\begin{aligned} \sum _{k=1}^{\infty } \text{ Var } ( W_{k} e^{-\alpha T_{k}} \textbf{1}_{\{ W_{k} e^{-\alpha T_{k}} \le A \}} ) \le \sum _{k=1}^{\infty } \mathbb {E} ( W_{k}^2 ) \mathbb {E} ( e^{-2\alpha T_{k}} ) = \frac{r_0\mu _{W}^2}{\alpha } < \infty . \end{aligned}$$

The three inequalities above show that the conditions of the Three series Theorem hold. Now, $\lim _{t\rightarrow \infty } e^{-\alpha t} Y(t) = \sum _{k=1}^{\infty } W_{k} c e^{-\alpha T_{k}}>0$ (a.s.). Hence,

$$\begin{aligned} \lim _{t\rightarrow \infty } F_k(t) {\mathop {=}\limits ^{a.s.}} \frac{W_{k} e^{-\alpha T_{k}} }{\sum _{k'=1}^{\infty } W_{k'} e^{-\alpha T_{k'}}}. \end{aligned}$$

This completes the proof of the first statement of Proposition 3.

Next, to get the limit of $J_Y(t,t+\delta )$, let $a(t,\delta )=\sum _{k=1}^{\Pi (t)} \textbf{1}_{\{W_k(t+\delta -T_k)>0\}}$, the number of clones started before or at time *t* and alive at time $t+\delta$. Then,

25 $$\begin{aligned} J_Y(t,t+\delta ) = 1-\frac{a(t,\delta )}{a(t,0)+a(t+\delta ,0)-a(t,\delta )}. \end{aligned}$$

Now, for every $t,\delta \ge 0$, $a(t,\delta )$ satisfies the inequalities

26 $$\begin{aligned} \sum _{k=1}^{\Pi (t-f(t))} \textbf{1}_{\{W_k(t+\delta -T_k)>0\}} \le a(t,\delta ) \le \sum _{k=1}^{\Pi (t-f(t))} \textbf{1}_{\{W_k(t+\delta -T_k)>0\}} + \Pi (t)- \Pi (t-f(t)). \end{aligned}$$

Consider first the left-hand side, normalized by $\Pi (t-f(t))$. Then,

$$\begin{aligned}{} & {} \lim _{t\rightarrow \infty } \frac{1}{\Pi (t-f(t))} \sum _{k=1}^{\Pi (t-f(t))} \textbf{1}_{\{W_k(t+\delta -T_k)>0\}}\\{} & {} \quad = \lim _{\ell \rightarrow \infty } \frac{1}{\ell } \sum _{k=1}^{\ell } \textbf{1}_{\{W_k>0\}} \\{} & {} \qquad + \lim _{t\rightarrow \infty } \frac{1}{\Pi (t-f(t))} \sum _{k=1}^{\Pi (t-f(t))} (\underbrace{\textbf{1}_{\{W_k(t+\delta -T_k)>0\}} - \textbf{1}_{\{W_k>0\}}}_{\ge 0}). \end{aligned}$$

The first term in the right-hand side converges a.s. to $\mathbb {P}\{W_1>0\}$ by a Strong Law of Large Numbers for i.i.d. r.v.. For the second one, notice that

$$\begin{aligned}{} & {} \mathbb {P} \left\{ \frac{1}{\Pi (t-f(t))}\sum _{k=1}^{\Pi (t-f(t))} ( \textbf{1}_{\{W_k(t+\delta -T_k)>0\}} - \textbf{1}_{\{W_k>0\}} )> \epsilon \right\} \\{} & {} \quad = \sum _{\ell =0}^\infty \mathbb {P} \left\{ \sum _{k=1}^{\ell } ( \textbf{1}_{\{W_k(t+\delta -T_k)>0\}} - \textbf{1}_{\{W_k>0\}} )> \ell \epsilon \big \vert \Pi (t-f(t)) = \ell \right\} \mathbb {P} \left\{ \Pi (t-f(t)) = \ell \right\} \\{} & {} \quad \le \sum _{\ell =0}^\infty \frac{ \sum _{k=1}^\ell (\mathbb {P}\{W_k(t+\delta -T_k)>0 \big \vert \Pi (t-f(t)) = \ell \} - \mathbb {P}\{W_k>0\})}{\ell \epsilon }. \end{aligned}$$

Since $\mathbb {P}\{W_k(t+\delta -T_k)>0 \big \vert \Pi (t-f(t)) = \ell \} - \mathbb {P}\{W_k>0\}$ is a monotone function in *t* for every $k,\ell$, we can exchange limit and summation to obtain

$$\begin{aligned}{} & {} \lim _{t\rightarrow \infty }\mathbb {P} \left\{ \frac{1}{\Pi (t-f(t))}\sum _{k=1}^{\Pi (t-f(t))} ( \textbf{1}_{\{W_k(t+\delta -T_k)>0\}} - \textbf{1}_{\{W_k>0\}} )> \epsilon \right\} \\{} & {} \quad \le \sum _{\ell =0}^\infty \frac{ \sum \limits _{k=1}^\ell \lim \limits _{t\rightarrow \infty } \mathbb {P}\{W_k(t+\delta -T_k)>0 \big \vert \Pi (t-f(t)) = \ell \} - \mathbb {P}\{W_k>0\}}{\ell \epsilon } = 0. \end{aligned}$$

We conclude that, for every $\delta \ge 0$,

$$\begin{aligned} \lim _{t\rightarrow \infty } \frac{1}{\Pi (t)} \sum _{k=1}^{\Pi (t-f(t))} \textbf{1}_{\{W_k(t+\delta -T_k)>0\}} {\mathop {\rightarrow }\limits ^{\mathbb {P}}} \lim _{t\rightarrow \infty } \frac{R(t-f(t))}{R(t)} \mathbb {P}\{W_1>0\} = \mathbb {P}\{W_1>0\}. \end{aligned}$$

Proceeding similarly, we show that the right-hand side of (26) normalized by $\Pi (t)$ also converges in probability to $\mathbb {P}\{W_1>0\}$. Therefore, for every $\delta \ge 0$, $a(t,\delta )/\Pi (t) {\mathop {\longrightarrow }\limits ^{\mathbb {P}}} \mathbb {P}\{W_1>0\}$ as $t\rightarrow 0$. We finally deduce from (25) that $J_Y(t,t+\delta ){\mathop {\longrightarrow }\limits ^{\mathbb {P}}}0$ as $t\rightarrow \infty$. Next, to prove the limit of $BC_Y(t,t+\delta )$, we write

$$\begin{aligned} BC_Y(t,t+\delta ) = \sum _{k=1}^{\infty } \frac{c W_k \vert e^{-\alpha T_k} -e^{-\alpha (T_k-\delta )}\vert \vert 1+o(1) \vert }{e^{-\alpha t}Y(t)+e^{-\alpha t}Y(t+\delta )} \textbf{1}_{\{T_k\le t+\delta \}}. \end{aligned}$$

Using previous results, we show that

$$\begin{aligned} \lim _{t\rightarrow \infty } BC_Y(t,t+\delta ) {\mathop {=}\limits ^{a.s.}} \frac{ \sum _{k=1}^{\infty } c W_k \vert e^{-\alpha T_k} -e^{-\alpha (T_k-\delta )}\vert }{\lim _{t\rightarrow \infty } e^{-\alpha t}Y(t)+\lim _{t\rightarrow \infty }e^{-\alpha t}Y(t+\delta )}, \end{aligned}$$

from which the result follows. To establish the limit of the Bhattacharyya coefficient, notice that for every $\delta \ge 0$

$$\begin{aligned} \lim _{t\rightarrow \infty } B_Y(t,t+\delta )&=\sum _{k=1}^\infty \sqrt{ \frac{\lim \limits _{t\rightarrow \infty } e^{-\alpha t} Z^{(k)}_k(t-T_k) \textbf{1}_{\{k \le \Pi (t+\delta )\}}}{\lim _{t\rightarrow \infty } e^{-\alpha t}Y(t)} }\\&\quad \times \sqrt{\frac{\lim \limits _{t\rightarrow \infty } e^{-\alpha (t+\delta )} Z^{(k)}_k(t+\delta -T_k) \textbf{1}_{\{k \le \Pi (t+\delta )\}}}{\lim _{t\rightarrow \infty }e^{-\alpha t}Y(t+\delta )} } \\&{\mathop {=}\limits ^{a.s.}}\sum _{k=1}^\infty \sqrt{\frac{W_k e^{-\alpha T_k}}{\sum _{k'=1}^\infty W_{k'} e^{-\alpha T_{k'}}}}\sqrt{\frac{W_k e^{-\alpha T_k}}{\sum _{k'=1}^\infty W_{k'} e^{-\alpha T_{k'}}}} \quad {\mathop {=}\limits ^{a.s.}} \ 1, \end{aligned}$$

To establish the limit of the proportion of similarity $P_Y(t,t+\delta )$, we have that

$$\begin{aligned} \lim _{t\rightarrow \infty } P_Y(t,t+\delta ) {\mathop {=}\limits ^{a.s.}} 1 - \frac{1}{2} \sum _{k=1}^{\infty } \bigg \vert \frac{W_k e^{-\alpha T_k}}{\sum _{k'=1}^\infty W_{k'} e^{-\alpha T_{k'}}} - \frac{W_k e^{-\alpha (T_k-\delta )}}{\sum _{k'=1}^\infty W_{k'} e^{-\alpha (T_k-\delta )}} \bigg \vert , \end{aligned}$$

and the result follows, completing the proof.

### Proof of Proposition 4

By assumption, $\lim _{t \rightarrow \infty } R(t) = \infty$. Therefore, $\Pi (t) {\mathop {\longrightarrow }\limits ^{a.s.}} \infty$ as $t\rightarrow \infty$, and the immigration process a.s. generates an infinite sequence $\{\alpha _k\}_{k=1}^\infty$. Let $\alpha _{max} = \inf \{x\ge 0: H^+(x)=1\}$ denote the upper bound of the support of the conditional distribution of $\alpha _1$, given $\alpha _1>0$. Since $\mathbb {P} \{ \alpha _1>0 \}>0$ by assumption, we deduce that $\alpha _{max}>0$. Now, $H^+(\cdot )$ is assumed to be absolutely continuous. Hence, $\alpha _k < \alpha _{\max }$ a.s., and $\mathbb {P}\{ \alpha _{k'}> \max (0,\alpha _{k}) \}>0$, $k'=k+1,k+2\cdots$. Therefore, $\sum _{k'=k+1}^\infty \mathbb {P}\{ \alpha _{k'} > \max (0,\alpha _{k}) \} = \infty$. Since the r.v. $\{\alpha _k\}_{k=1}^\infty$ are i.i.d., we deduce from the second Borel–Cantelli Lemma that there exists a.s. an infinite subsequence $\{\alpha _{k_\ell }\}_{\ell =1}^\infty \subseteq \{\alpha _{k'}\}_{k'=k+1}^\infty$ such that $\alpha _{k_\ell }>\max (0,\alpha _{k})$, $\ell =1,2\ldots$. Then, for every $k=1,2\ldots$, $e^{-\alpha _k t} Z^{(k)}_k(t) {\mathop {\longrightarrow }\limits ^{a.s.}}W_k$ using the assumption that $\int _0^\infty g_k(x)^\delta dx <\infty$ for some $\delta >1$ (see Assumption (A4)). The sequence $\{W_{k_\ell }\}_{\ell =1}^\infty$ is i.i.d and such that $\mathbb {P}\{ W_{k_\ell }>0\} >0$, $\ell =1,2\ldots$. Hence, a.s., $\exists \ell _+ \in \{1,2\ldots \}$ such that $W_{k_{\ell _+}}>0$. We deduce that

$$\begin{aligned} 0\le & {} \lim _{t \rightarrow \infty } F_k(t) \le \lim _{t \rightarrow \infty } \frac{Z^{(k)}_k(t-T_k)}{Z^{(k_{\ell _+})}_{k_{\ell _+}}(t-T_{k_{\ell _+}})}\\= & {} \lim _{t \rightarrow \infty } \left( \frac{ e^{-\alpha _k(t-T_k)} Z^{(k)}_k(t-T_k)}{e^{-\alpha _{k_{\ell _+}}(t-T_{k_{\ell _+}})} Z^{(k_{\ell _+})}_{k_{\ell _+}}(t-T_{k_{\ell _+}})} \frac{ e^{\alpha _k(t-T_k)} }{e^{\alpha _{k_{\ell _+}}(t-T_{k_{\ell _+}})} } \right) \\= & {} \frac{ W_k}{W_{k_{\ell _+}}} \lim _{t \rightarrow \infty } \frac{ e^{\alpha _k(t-T_k)} }{e^{\alpha _{k_{\ell _+}}(t-T_{k_{\ell _+}})}} \ {\mathop {=}\limits ^{a.s.}} \ 0. \end{aligned}$$

This proves the first claim of Prop. 4 and its second one by setting $k' = k_{\ell _+}$.

### Proof of Proposition 5

We have $M^*(s) = G^*(s)M = 2(1-p_0) G^*(s) Q$. Hence, the largest eigenvalue of $M^*(s)$ is $\rho ^*(s)=2(1-p_0) G^*(s) \rho ^*_Q$ where $\rho ^*_Q$ denotes the largest eigenvalue of *Q*. Now, it can be shown that *Q* is a stochastic matrix. Therefore, $\rho ^*_Q=1$, and the associated right eigenvector is $\textbf{u}=(\frac{1}{K},\dots ,\frac{1}{K})$. Next, since $\alpha$ is a solution of the equation $\rho ^*(\alpha ) = 1$, we have that $G^*(\alpha ) = 1/(2(1-p_0))$ and we deduce that $\alpha = G^{*,-1} \big (1/(2(1-p_0))\big ) >0$ because the Laplace transform of a real-valued positive function is strictly decreasing. Using results from Sect. 3.6, we conclude that $e^{-\alpha t}{} \textbf{Z}(t) {\mathop {\longrightarrow }\limits ^{a.s.}} W\textbf{v}$ where $\textbf{v}$ is the left eigenvector of $M^*(\alpha ) = 2(1-p_0) G^*(\alpha ) Q$ and the left eigenvector of *Q* associated with its unit eigenvalue which satisfies $u_1 v_1+\cdots +u_K v_K= \frac{1}{K}(v_1+\cdots +v_K) = 1$.
